# Innovative Delivery of siRNA to Solid Tumors by Super Carbonate Apatite

**DOI:** 10.1371/journal.pone.0116022

**Published:** 2015-03-04

**Authors:** Xin Wu, Hirofumi Yamamoto, Hiroyuki Nakanishi, Yuki Yamamoto, Akira Inoue, Mitsuyoshi Tei, Hajime Hirose, Mamoru Uemura, Junichi Nishimura, Taishi Hata, Ichiro Takemasa, Tsunekazu Mizushima, Sharif Hossain, Toshihiro Akaike, Nariaki Matsuura, Yuichiro Doki, Masaki Mori

**Affiliations:** 1 Department of Surgery, Gastroenterological Surgery, Graduate School of Medicine, Osaka University, Suita, Japan; 2 Research Fellow of Japan Society for the Promotion of Science, Tokyo, Japan; 3 Nakanishi Gastroenterological Research Institute, Sakai, Japan; 4 Department of Biomolecular Engineering, Graduate School of Bioscience and Biotechnology, Tokyo Institute of Technology, Yokohama, Japan; 5 Biomaterials Center for Regenerative Medical Engineering, Foundation for Advancement of International Science, Tsukuba, Japan; 6 Osaka Medical Center for Cancer and Cardiovascular Diseases, Osaka, Japan; University of Utah, UNITED STATES

## Abstract

RNA interference (RNAi) technology is currently being tested in clinical trials for a limited number of diseases. However, systemic delivery of small interfering RNA (siRNA) to solid tumors has not yet been achieved in clinics. Here, we introduce an *in vivo* pH-sensitive delivery system for siRNA using super carbonate apatite (sCA) nanoparticles, which is the smallest class of nanocarrier. These carriers consist simply of inorganic ions and accumulate specifically in tumors, yet they cause no serious adverse events in mice and monkeys. Intravenously administered sCA-siRNA abundantly accumulated in the cytoplasm of tumor cells at 4 h, indicating quick achievement of endosomal escape. sCA-survivin-siRNA induced apoptosis in HT29 tumors and significantly inhibited *in vivo* tumor growth of HCT116, to a greater extent than two other *in vivo* delivery reagents. With innovative *in vivo* delivery efficiency, sCA could be a useful nanoparticle for the therapy of solid tumors.

## Introduction

Remarkable advances have been made in clinical cancer therapy, using monoclonal antibodies and small molecule inhibitors to treat various tumor types [1–2]. One of the most important basic biological advances has been the discovery of RNA interference (RNAi) [3]. Small interfering RNAs (siRNAs) are expected to have wide therapeutic applications in human disease because of their ability to silence any gene with a known sequence and because of their functional activity [4]. Local therapy using naked siRNA products targeting the eye, skin, and lung are progressing through clinical trials [5]. In contrast to local administration, the systemic delivery of siRNA requires an appropriate vehicle because naked siRNA undergoes rapid degradation in the blood stream [6]. Nanoparticles are one possible systemic delivery tool for siRNA. A common feature of recent RNA carriers with diameters of 45–300 nm, which include liposomes, polymers, and atelocollagen, is that *in vivo* systemic administration often results in accumulation in the liver, spleen, kidney, or lung [7–9]. Accordingly, several siRNA drugs have been developed to deliver siRNAs to the liver and kidney, and are currently being tested as therapies for hypercholesterolemia, acute kidney injury, and transthyretin mediated amyloidosis [10].

However, RNAi technology for solid tumors, especially in systemic administration, still faces numerous hurdles [11]. Nanoparticles theoretically permit passive targeting of tumors *in vivo* because tumor tissues have enhanced permeability and retention characterized by increased microvasculature leakage and impaired lymphatic function [12]. Remarkable experimental progress has been made in designing nanoparticle carriers, and several nanomedicines containing anti-cancer reagents have been successfully developed against human tumors [13]. However, an ideal system that provides sufficient intracellular delivery of siRNA to the target tumor cells remains unrealized in clinical cancer therapy. Indeed, only four siRNA products have advanced to Phase I trials in solid tumors: the polyethylene glycol (PEG)ylated polymer CALAA-01 particle and the lipid-based nanoparticles TKM-PLK1, ALN-VSP, and Atu027 [10].

The process for generating carbonate apatite is quite simple, involving a mixture of the inorganic ions CO_3_^2−^, Ca^2+^, and PO_4_^3−^. The resulting nanoparticles are highly stable at the physiological pH of 7.4 and quickly degradable at acidic pH in the endosomal compartments of tumor cells. In mammalian cells, carbonate apatite provides approximately 10–100-fold higher transfection efficiency of DNA *in vitro* compared with Lipofectamine 2000 (Lp) or calcium phosphate precipitation[14]. *In vitro* studies have also shown that carbonate apatite is an efficient carrier for siRNA into mammalian cells and allows sufficient knockdown of gene expression of cyclin B1[15]. However, the feature of *in vivo* systemic delivery of siRNA to solid tumors by carbonate apatite is largely unknown.

A brief sonication of original carbonate apatite with an appropriate stabilizing agent resulted in the creation of 10∼20 nm nanoparticles in majority. In order to show the *in vivo* distribution of intravenously administered sonicated carbonate apatite nanoparticles, we performed comparative studies with two currently available systemic *in vivo* siRNA delivery systems, Invivofectamine 2.0 (IF) and AteloGene (Atelo). The IF reagent is lipid-based and the latest innovation for systemic siRNA delivery *in vivo* [16, 17]. Atelo consists of a highly purified type I collagen that is free of immunogenic telopeptides [18, 19]. To examine the therapeutic potential and to obtain functional evidence of the sonicated carbonate apatite system, we administered the nanoparticles with survivin siRNA to pre-established HCT116 and HT29 tumor models in mice. Owing to its distinctly innovative delivery efficiency and anti-tumor activity *in vivo*, we designated the nanoparticle as super carbonate apatite (sCA).

## Results

### Properties of sCA technology

**Fig 1:**
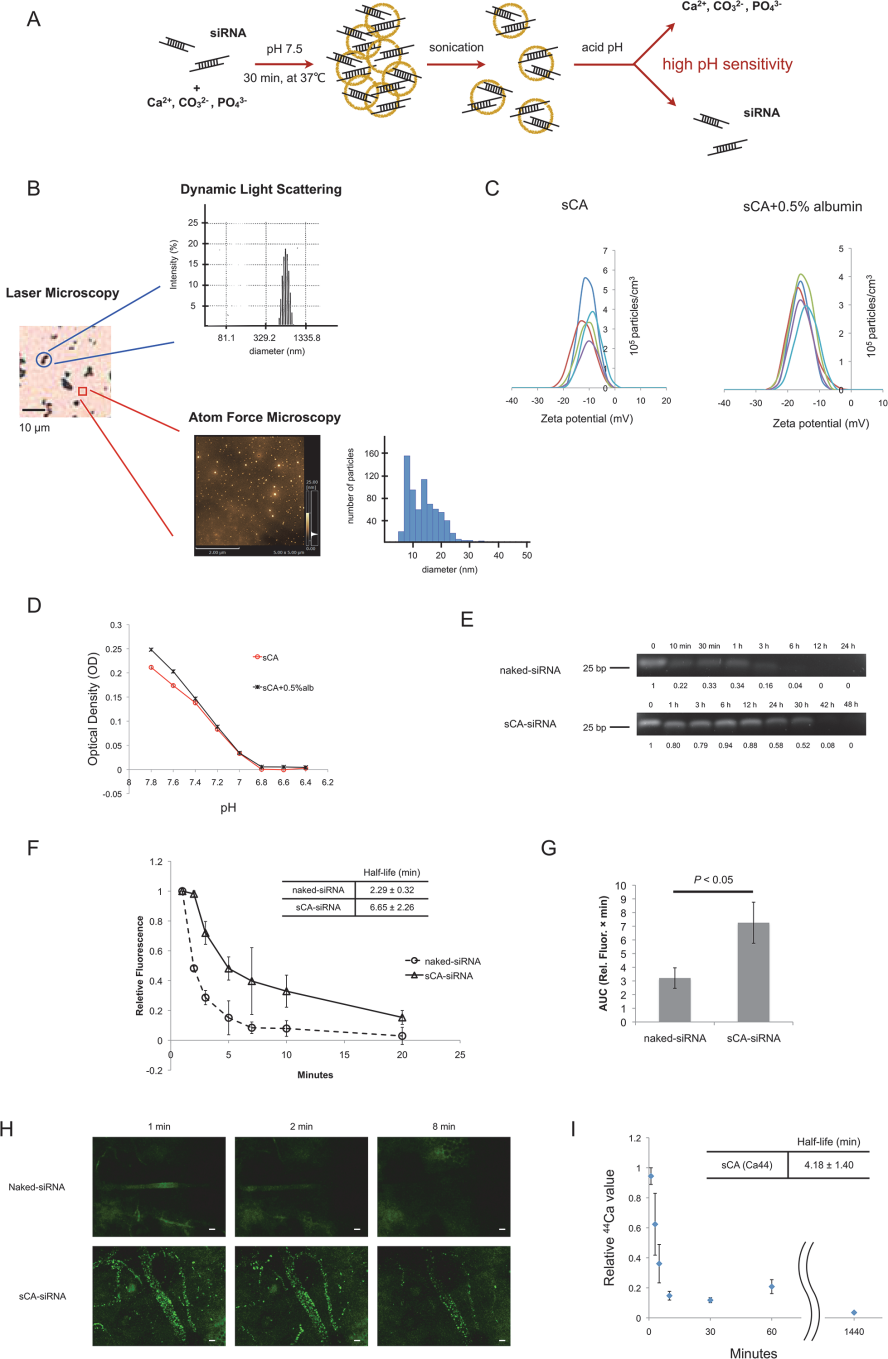
Characterization of super carbonate apatite (sCA). (A) Schematic presentation of sCA incorporating siRNA. The straightforward generation process involves a mixture of the inorganic ions CO_3_^2-^, Ca^2+^, and PO_4_^3-^ and incubation at 37°C for 30 min. After sonication, the sCA nanoparticles can be degraded at acidic pH to release the incorporated siRNA compounds. (B) Size distribution of sCA+0.5% albumin nanoparticles was measured by laser microscopy, dynamic light scattering (DLS) and atomic force microscopy (AFM). DLS showed the size of the larger nanoparticles was 653 nm. AFM analyses were performed to visualize the smaller nanoparticles, on the smooth surface excluding the larger nanoparticles. The smaller nanoparticle size ranged from 7 to 50 nm (mean ± SD: 14.8 ± 5.66 nm). The percentage of smaller nanoparticles among the whole sCA+0.5% albumin nanoparticles was calculated to be 99.7% in particle numbers. (C) Zeta potential (mV) of sCA (left) and sCA+0.5% albumin (right) were found to be-10.69 ± 2.19 mV and-16.02 ± 0.89 mV, (mean ± SD; n = 5), when incubated by 4mM Ca^2+^-supplemented bicarbonated buffer. (D) pH sensitivity of sCA, sCA+0.5% albumin. The optical density (OD) value of sCA, or sCA+0.5% albumin decreased as pH value decreased. (E) *Ex vivo* half-lives of naked-siRNA and sCA-siRNA (40 μg) in mouse serum, incubated at 37°C over a period of up to 48 h. (F)(G)(H) *In vivo* circulation properties of naked-siRNA and sCA-siRNA, following tail-vein intravenous injection (80 μg siRNA for naked-siRNA, 40 μg siRNA for sCA-siRNA). (F) Relative fluorescence intensity curves and *in vivo* half-lives (mean ± SD; n = 3). (G) Area under the curve values over 20 min after injection (n = 3, Wilcoxon rank test). (H) Representative ear microvasculature snap-shots at 1, 2, and 8 min; Green: 6-FAM labeled siRNA. Scale bar, 20 μm. (I) *In vivo* half-life in blood circulation of sCA nanoparticle. The original Ca was replaced to isotope ^44^Ca during the manufacture, and the concentration of ^44^Ca was measured by ICP-MS (n = 3, mean ± SD: 4.18 ± 1.40 min).

The super carbonate apatite (sCA) nanoparticles consisting of inorganic ions (*i.e.*, CO_3_^2-^, Ca^2+^, and PO_4_^3-^) were degradable at acidic pH, leading to the release of incorporated siRNA compounds (Fig. 1A). After sonication, the nanoparticle size ranged from 5 to 30 nm (mean ± SD: 10.50 ± 5.01 nm) when measured by atomic force microscopy (AFM) (S1A Fig.), but the sCA precipitated within 1 min (S1B Fig.). To maintain the sCA nanoparticles to be disrupted for *in vivo* use, we added 0.5% albumin during the sonication and it prevented precipitation at 10 min. Investigation using laser microscopy, dynamic light scattering (DLS) and atomic force microscopy (AFM) revealed that the size of sCA+0.5% albumin nanoparticles displayed two distinct populations after sonication (Fig. 1B). DLS analysis showed that mean size of the larger nanoparticles was 653 nm and AFM demonstrated that the smaller nanoparticle size ranged from 7 to 50 nm (mean ± SD: 14.8 ± 5.66 nm). The percentage of smaller nanoparticles was calculated to be 99.7% in particle numbers. The zeta potential of sCA and sCA+0.5% albumin were found to be-10.69 ± 2.19 mV and-16.02 ± 0.89 mV, respectively (mean ± SD; n = 5) (Fig. 1C). sCA and sCA+0.5% albumin were pH sensitive. Both of them were degradable when pH decreased from 7.8 to 6.8 (Fig. 1D). Naked-siRNA was degraded to approximately 20% of the original amount in only 10 min when incubated in mouse serum at 37°C (Fig. 1E). In contrast, siRNA incorporated into sCA was highly stable in the serum; the *ex vivo* half-life was approximately 30 h, suggesting that sCA was resistant to serum nucleases. As for the *in vivo* stability, fluorescence intensity of naked-siRNA and sCA-siRNA was monitored by multiphoton live imaging at the mice ear microvasculature following intravenous injection. Relative fluorescence intensity curves, half-lives, area under the curves (AUCs), are shown in Fig. 1F and G. sCA showed a 2.9-fold improvement of circulation half-life of siRNA and 2.3-fold increase in AUC over 20 min compared to naked-siRNA. The representative time-lapse images of ear microvasculature snap-shots showed that sCA increased the blood residence time of siRNA (Fig. 1H). To investigate the *in vivo* half-life of sCA nanoparticle itself, we replaced the Ca to isotope ^44^Ca during the manufacture, and performed inductively coupled plasma mass spectrometry (ICP-MS) to measure the concentration of ^44^Ca in blood serum following the intravenous injection of sCA. The results were consistent with the results provided by multiphoton live imaging (Fig. 1I).

### *In vitro* transfection efficiency of sCA

**Fig 2:**
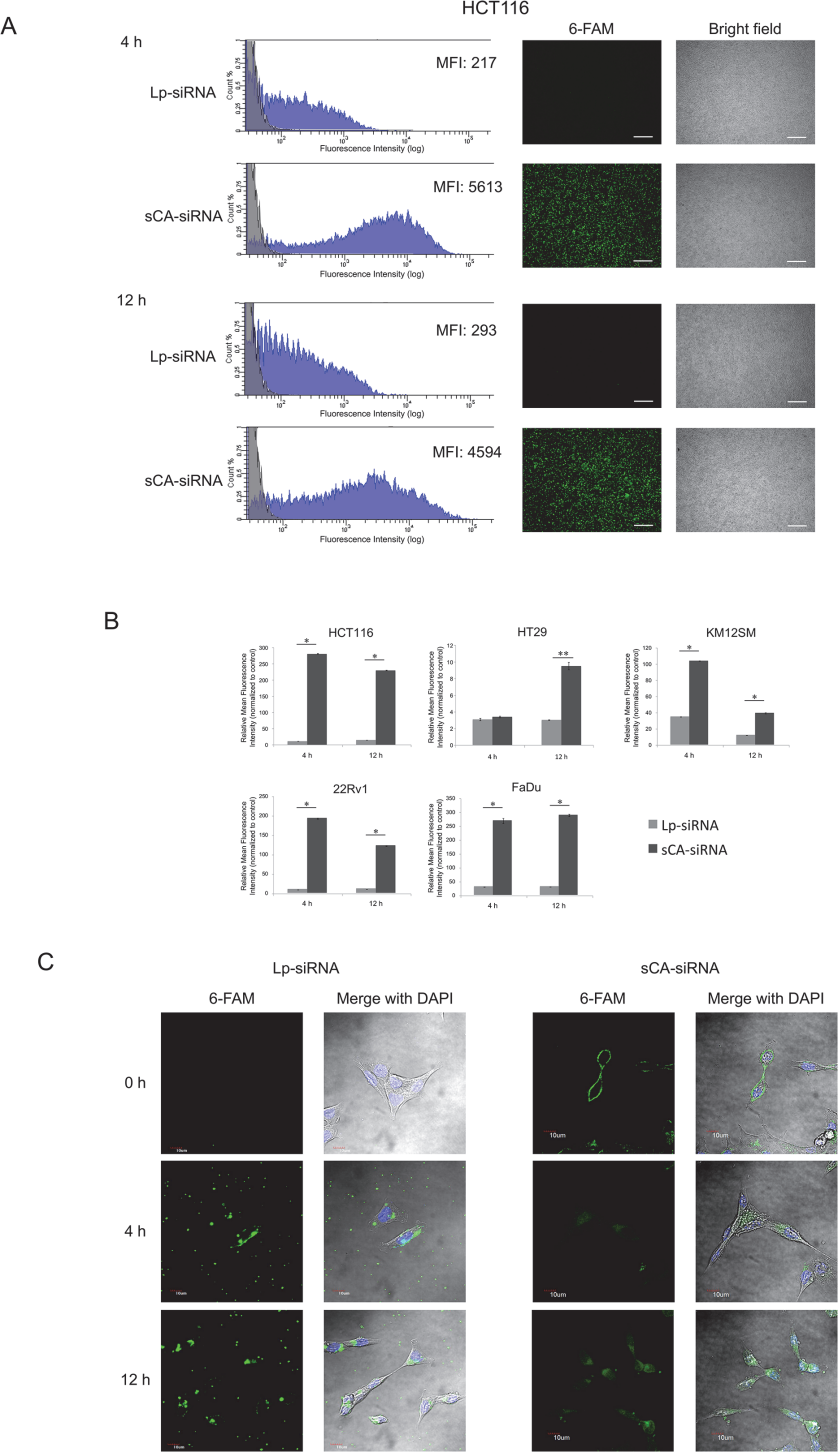
*In vitro* transfection efficiency by lipofectamine 2000 (Lp) or sCA. (A) HCT116 was examined for transfection efficiency of 6-FAM labeled siRNA (20 pmol/well) by Lp or sCA. Cells were analyzed with flow cytometry at 4 and 12 h and then further observed by fluorescence microscopy. Bright field images are shown in the right panels. MFI; mean fluorescence intensity. Scale bars, 300 μm. (B) The relative MFI of Lp-siRNA or sCA-siRNA treated cells. Data represent mean ± SEM. *P = 0.0304, **P = 0.0294 (n = 4, Wilcoxon rank test). (C) Uptake behavior of Lp-siRNA or sCA-siRNA in HCT116 cells analyzed by confocal laser scanning microscopy at 4 and 12 h. Merged panels show fluorescent siRNA (green) and DAPI nuclear staining (blue). Scale bars, 10 μm.

Superior *in vitro* transfection efficiency by sCA relative to Lipofectamine 2000 (Lp) was confirmed in different cell types. Quantitative flow cytometry analyses and fluorescence images showed that sCA carried 6-FAM labeled control siRNA into the human colon cancer cell lines (HCT116, HT29, KM12SM), the 22Rv1 human prostate cancer cell line, and the FaDu human head and neck cancer cell line (Fig. 2A, B, S2 Fig.). At both 4 and 12 h, the mean fluorescence intensity (MFI) for the cells treated with sCA-siRNA was significantly higher than that with Lp-siRNA in these cell lines (Fig. 2B, *P = 0.0304, **P = 0.0294). The differential cellular uptake behavior was further analyzed in HCT116 with high-resolution confocal microscopy. Fluorescence signal was deposited only as several spots in the cytoplasm by Lp at 4 and 12 h, while green fluorescence of the sCA-siRNA appeared in the cytoplasm at 4 h and subsequently accumulated throughout the cytoplasm at 12 h (Fig. 2C).

### *In vivo* bio-distribution of sCA in normal and tumor tissues

**Fig 3:**
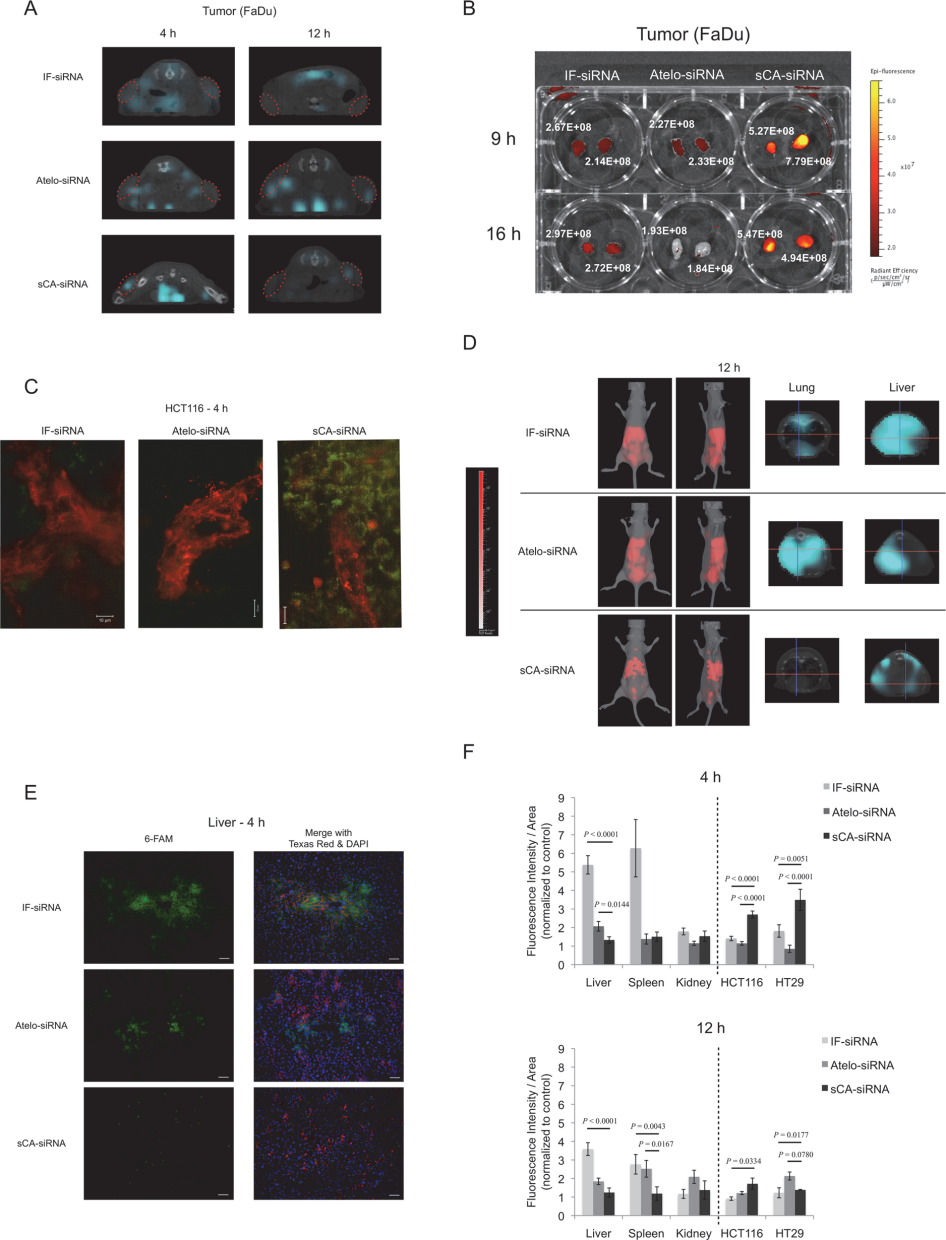
Bio-distribution of sCA-siRNA in normal and tumor tissues. (A) CT imaging of Alexa Fluor 750 labeled siRNA in FaDu tumors at 4 and 12 h. The red dashed circles indicate tumors. (B) *Ex vivo* imaging at 9 and 16 h comparing sCA with IF and Atelo. (C) Detection of siRNA in HCT116 tumor at 4 h by light sheet fluorescence microscopy. The microvasculature was visualized as red signals by intravenous injection of Texas Red conjugated dextran. Green: 6-FAM labeled siRNA. Scale bar, 10 μm. (D) IVIS spectrum CT imaging 12 h after treatment. Mice were treated with 40 μg of Alexa Fluor 750 labeled siRNA delivered with Invivofectamine 2.0 (IF), AteloGene (Atelo), or sCA by intravenous administration via tail vein. Left panel: whole mouse imaging; right panel: CT scan images of lung and liver. (E) Detection of 6-FAM labeled siRNA distribution by fluorescence microscopy in liver 4 h after intravenous injection. The microvasculature was visualized with red signals by intravenous injection of Texas Red conjugated dextran. Left, 6-FAM labeled siRNA; right, merged images of the left panel and Texas Red stained blood vessels with DAPI. Scale bar, 50 μm. (F) Quantitative analysis of fluorescence intensity at 4 and 12 h. Fluorescence of 6-FAM was calculated as fluorescence intensity/area and normalized against that of control sections. Data represent mean ± SEM. Liver: n = 12∼15 fields of view (FOV) from 4 mice; Spleen: n = 12∼20 FOV from 4 mice; Kidney: n = 8∼16 FOV from 4 mice; HCT116: n = 21∼24 FOV of 6 tumors; HT29: n = 8∼24 FOV of 6 tumors (Wilcoxon rank test).

We then examined the *in vivo* delivery of Alexa Fluor 750 fluorescently labeled siRNA by sCA to pre-established subcutaneous tumors in nude mice compared with other types of *in vivo* systemic delivery vehicles: Invivofectamine 2.0 (IF: liposome for *in vivo* use) and AteloGene (Atelo). The binding affinity of siRNA to sCA nanoparticles was calculated to be 40.6 ± 2.9% (mean ± SD, n = 4). Mice bearing FaDu tumors were intravenously injected with IF-siRNA, Atelo-siRNA, or sCA-siRNA (40 μg for each). Three-dimensional *in vivo* imaging CT analysis revealed little fluorescence in the tumors treated with IF-siRNA and Atelo-siRNA at 4 h, while sCA-siRNA fluorescence accumulated at the center of the tumors at 4 h (Fig. 3A). At 12 h, reduced fluorescence signal remained in the tumors of the sCA-siRNA treated mice. *Ex vivo* imaging analysis demonstrated that sCA-siRNA exhibited higher radiant efficiency in the tumors at 9 and 16 h compared with the other two systems (Fig. 3B). Furthermore, a much higher radiant efficiency of sCA-siRNA was detected in the tumors 3 h after injection (S3A Fig.). Time course studies revealed that the uptake of sCA-siRNA occurred as early as 90 min (S3B Fig.). Light sheet fluorescence microscopy showed that the green fluorescence of sCA-siRNA was found in HCT116 cells 4 h after intravenous injection, whereas such signals were scarcely detected by IF-siRNA and Atelo-siRNA treatment (Fig. 3C). Studies of serial sections of sCA treated HCT116 tumors revealed that fluorescence of sCA-siRNA spread throughout the cytoplasm of tumor cells, which is highly indicative of early achievement of endosomal escape (S3C Fig.).

The amount of Alexa Fluor 750 fluorescently labeled siRNA administered was adjusted to the equivalent of 40 μg across three *in vivo* delivery systems. As shown in Fig. 3D, IVIS spectrum CT revealed that a diffuse intense fluorescence accumulated in liver but not in lung 12 h after tail vein injection of IF-siRNA. High fluorescence accumulated in lung and liver of Atelo-siRNA treated mice (Atelo-siRNA). Compared with the other two systems, the sCA-siRNA treated mice exhibited an accumulation of nodular fluorescence in liver, but no deposit was found in lung. Liver uptake was the most notable with all three vehicles. When we examined the distribution of sCA nanoparticle *in situ* using the short wavelength 6-FAM, a microscopic survey revealed that green fluorescence derived from IF-siRNA or Atelo-siRNA was noted in the cytoplasm of hepatic cells, mainly around central veins, at 4 and 12 h. However, the uptake of sCA-siRNA into hepatocytes was a relatively rare event (Fig. 3E, S3D Fig.). In spleen, IF-siRNA and Atelo-siRNA accumulated at both 4 and 12 h (S3E Fig.). The fluorescence was almost negligible in kidney sections at 4 and 12 h across the three systems (S3F Fig.).

Quantitative analyses using multi-slice sections indicated that sCA was delivered to normal organs to a lesser extent. Thus, uptake of sCA-siRNA in liver and spleen was significantly decreased compared with IF-siRNA and Atelo-siRNA (Fig. 3F). In the kidney, there was no significant difference in fluorescence levels among the three systems. By contrast, there was a significant increase in fluorescence intensity in the sCA-siRNA treated HCT116 or HT29 tumors, especially at the early time point of 4 h.

### Anti-tumor effects and functional evidence of sCA-survivin-siRNA

**Fig 4:**
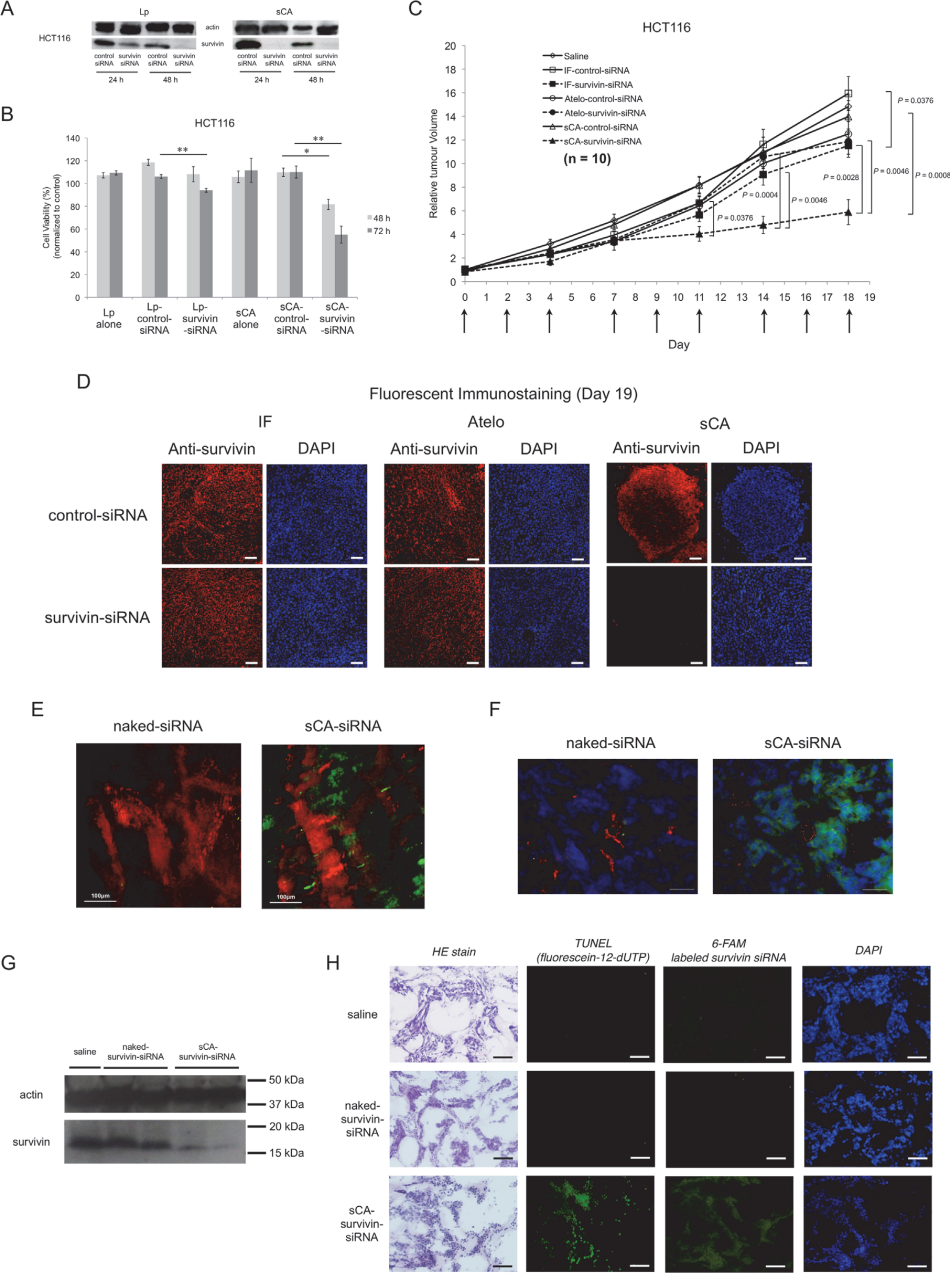
Anti-tumor effects and functional evidence of sCA-survivin-siRNA in HCT116 and HT29 solid tumor models. (A) For western blot analysis, HCT116 cells were seeded into 6-well plates and transfected with 100 pmol/well of either control or survivin siRNA by Lp (Lipofectamine 2000) or sCA. Actin blots served as loading controls. (B) For proliferation assays, cells were uniformly seeded into 96-well plates (1 × 10^4^ cells/well), and 5 pmol/well siRNA was used. Cell viability was examined at 48 and 72 h by WST-8 assay. Data represent mean ± SEM. *P = 0.0294, **P = 0.0304 (n = 4, Wilcoxon rank test). (C) *In vivo* tumor growth. Each vehicle, carrying 15 μg of control siRNA or survivin siRNA, was administered by intravenous injection to mice with HCT116 tumors. Data represent the mean ± SEM (n = 10 tumors, Wilcoxon rank test). (D) Immunostaining of survivin in the tumor tissues on day 19. Scale bar, 50 μm. (E) *In vivo* live imaging of sCA-siRNA (6-FAM labeled) in HT29 tumor by multiphoton microscopy at 90 min. (F) Fluorescent detection of naked-siRNA (6-FAM labeled) or sCA-siRNA (6-FAM labeled) in the HT29 tumor at 4 h. Green: 6-FAM labeled siRNA; Red: microvasculature; Blue: DAPI stained nuclei. Scale bar, 50 μm. (G) Mice were administered with 40 μg of naked-survivin-siRNA or sCA-survivin-siRNA on days 0, 1, and 2. Tumors were removed on day 3, and western blot analysis for survivin was performed. (H) Many tumor cells treated with sCA-survivin-siRNA (6-FAM labeled) had condensed nuclei and were positive for TUNEL assay. Scale bar, 50 μm.

Survivin is a member of the inhibitor of apoptosis family and performs multiple functions, including cell cycle regulation and inhibition of cell death [20, 21]. *In vitro* studies showed that survivin protein in HCT116 decreased more dramatically with sCA-survivin-siRNA transfection compared to Lp-survivin-siRNA (Fig. 4A). Accordingly, sCA treatment more drastically reduced the growth of HCT116 cells at 48 and 72 h post-transfection (*P* = 0.0294, 0.0304, respectively, Fig. 4B). sCA vehicle alone, 2-fold-, or 4-fold quantity of sCA was not harmful to HCT116 cells (data not shown). In the mouse therapeutic model of pre-established HCT116 tumors, IF, Atelo, or sCA incorporating a relatively low dose of survivin siRNA (15 μg per injection) was intravenously injected through the tail vein three times per week for 3 weeks. As shown in Fig. 4C, much stronger growth inhibition was noted in sCA-survivin-siRNA treated mice compared with other systems. On day 18, the tumor volume of mice treated with sCA-survivin-siRNA was significantly smaller than in mice treated with sCA-control-siRNA (*P* = 0.0008), IF-survivin-siRNA (*P* = 0.0028), or Atelo-survivin-siRNA (*P* = 0.0046). On day 19, mice were sacrificed, and fluorescent immunostaining of the survivin protein in tumor tissues was performed. Only sCA-survivin-siRNA, but not other delivery systems, knocked down survivin protein expression (Fig. 4D). Immune-associated non-specific effects were not observed by the sequence of survivin siRNA used in this study (S4 Fig.).

To further verify the *in vivo* efficacy of sCA, we conducted additional functional assays in the pre-established HT29 colon cancer model. Multiphoton microscopy revealed that the sCA-siRNA, but not the naked-siRNA extravasated from tumor vessels as early as 90 min after i.v. injection (Fig. 4E), which is possibly because of enhanced permeability and retention effects. An extensive range of green fluorescently labeled siRNA delivered by sCA was present in tumor cells at 4 h under fluorescence microscopy (Fig. 4F). Three repeated injections of sCA-survivin-siRNA led to a considerable decrease in survivin protein expression in solid tumors (Fig. 4G). Histological examination indicated that many tumor cells treated with sCA-survivin-siRNA had condensed nuclei, which is one of the typical morphological features of apoptosis. The fluorescence of 6-FAM labeled survivin siRNA and 12-dUTP apoptotic signals were present in the tumors treated with sCA-survivin-siRNA but not with naked-survivin-siRNA or saline (Fig. 4H). These findings indicated that the sCA-survivin-siRNA functioned efficiently in the tumor cells *in vivo*.

### *In vivo* toxicity study on sCA in animals

**Fig 5:**
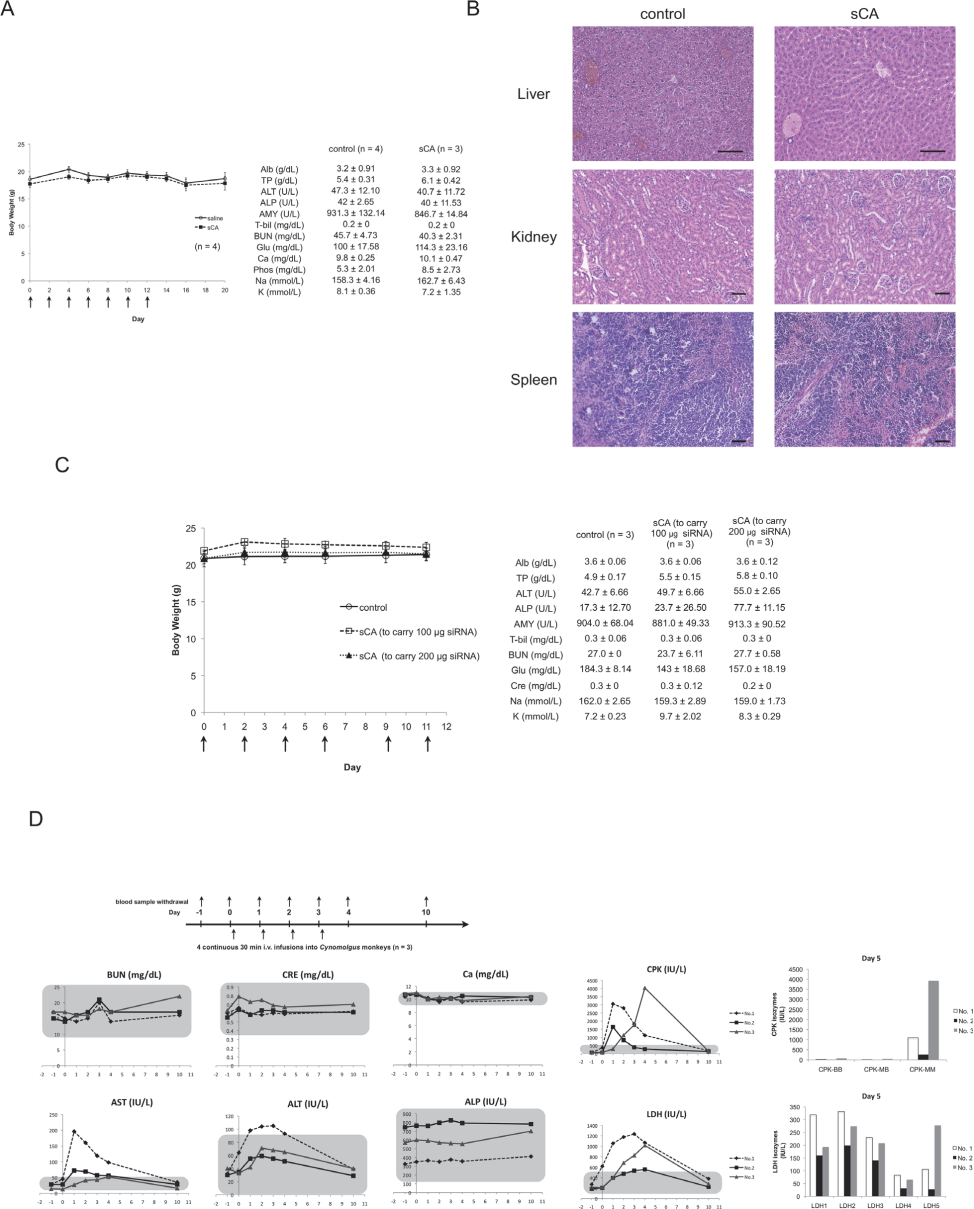
*In vivo* toxicity study on sCA in animals. sCA nanoparticles alone were i.v. administered to mice on days 0, 2, 4, 6, 8, 10, and 12. The amount of sCA per injection corresponds to that needed to carry 40 μg of siRNA. On day 20, mice were sacrificed, their blood was collected for serum chemistry analysis and the organs were investigated by histological examination. (A) Body weight curves of mice and blood chemistry data for saline treated and sCA treated mice. (B) Hematoxylin and eosin-stained liver, kidney, and spleen sections. Scale bar, 50 μm. (C) Mice were treated by intravenous injection of 2.5-fold sCA (100 μg siRNA per injection) or 5-fold sCA (200 μg siRNA per injection) on days 0, 2, 4, 6, 9, and 11. On day 12, mice were sacrificed, and their blood was collected for serum chemistry analysis. Body weight curves and blood chemistry data showed no abnormal findings for saline treated and sCA treated mice. (D) Blood chemistry data from *Cynomolgus* monkeys on days 1∼4 and day 10 (n = 3). Monkeys received 30 min i.v. infusion of sCA on days 0, 1, 2, and 3. The amount of sCA per injection corresponds to that carrying 0.4 mg/kg siRNA. Area within the gray rectangle indicates normal range (mean ± 2 SD). Isozyme analysis of CPK and LDH from blood sample on day 5 is shown together. Each isozyme is derived from the specific organs as follows; CPK-BB: brain; CPK-MB: cardiac muscle; CPK-MM: skeletal muscle; LDH1–2: cardiac muscle, skeletal muscle, red blood cell, and kidney; LDH5: liver, skin and muscle.

For *in vivo* safety, we carried out a toxicity study by intravenous injection of sCA (the amount of sCA per injection corresponds to that needed to carry 40 μg of siRNA) to mice every two days, 7 times. We noted no mortality or body weight loss. Blood chemistry tests showed no physiologically significant difference between the saline and sCA treated groups (Fig. 5A). Hematoxylin and eosin stained sections of the liver, kidney and spleen showed that sCA caused no particular histological damage (Fig. 5B). There was no abnormality in body weight or blood chemistry when sCA was increased to the amount to carry 100 or 200 μg siRNA per injection, which was 2.5-fold and 5-fold greater amounts of sCA, respectiely (Fig. 5C). We carried out a chronic toxicity study by intravenous injection of sCA (the amount of sCA per injection corresponds to that needed to carry 40 μg of siRNA) to mice every two days, 9 times. At day 31, we harvested the kidney, urinary duct and bladder to investigate the existence of calculi using X-ray photography and stereomicroscope. As shown in S5 Fig., there were no calculi in the urinary organs treated with sCA compared to the control ones.

To aim the final goal of therapy of human tumors, we made up 70-fold sCA of that administered in mouse per injection and administered it to monkeys on 4 consecutive days (n = 3). Monitored items are listed in S1–3 Tables. Monkeys’ bodies were restricted for 30 min during i.v. injection of sCA. During and after injection, general condition of monkeys was nothing particular to note, and urine tests indicated no abnormalities. As shown in Fig. 5D, blood tests indicated no abnormalities except for the following 4 enzymes; one monkey (No.1) had an increase in serum AST with a peak on day 1 and serum ALT with a peak on day 3. All the monkeys had an increase in CPK level and isozyme analysis indicated that the majority was CPK-MM derived from skeletal muscle. Two monkeys (No.1 and No.3) had an increase in LDH levels with distinct isozyme distribution. These abnormal values recovered to the normal range on day 10.

## Discussion

Current systemic delivery of siRNA to solid tumors using nanoparticles still faces hurdles that limit the use of RNAi in clinics [8, 10, 11, 22–26 33–38]. These hurdles include (i) capture by the reticuloendothelial system (RES; *e.g.*, liver and spleen) [11, 22], (ii) limited penetration into tumor cells due to large particle size [23, 24], and (iii) lysosomal clearance of nanoparticles and siRNA in tumor cells [25, 26]. Here, in sCA, we show an efficient, simplified, and unique nanoparticle carrier that could surmount most of these challenges.

The potential of sCA as an *in vivo* delivery vehicle for RNAi with regard to *in vivo* bio-distribution and efficacy has not been evaluated. This is because the original carbonate apatite particles form into large aggregations (S6 Fig.). The aggregation at the early stage of crystallization is a common problem with the calcium phosphate precipitation method [27]. To prevent aggregation, Pittella *et al*. used PEGylation during the process of nanoparticle development, which enlarged the particle size to 100 nm [28]. It has also become clear that intravenously injected particles >100 nm in diameter are likely to be trapped by the RES in the liver and spleen, leading to degradation by activated monocytes and macrophages [11, 23, 24]. Instead of using PEGylation or complex modifications, we disrupted the aggregation of carbonate apatite particles by sonication, resulting in generation of 10∼20 nm sized sCA nanoparticles for *in vivo* use.

Laser microscopy and atomic force microscopy revealed that the majority of sCA for *in vivo* use was approximately 15 nm-sized nanoparticle. Therefore, along with RNA packaging nanoparticles [29, 30], sCA is the smallest class of nanoparticles compared with currently available nanoparticles, such as liposomes, micelles, polymers, and atelocollagen (S7 Fig.). The minimal size of sCA nanoparticles has an advantage not only in avoiding RES clearance but also in wide penetration of tumor tissues. Perrault *et al*. reported that 100 nm PEGylated gold nanoparticles accumulated only at the perivascular space but that 20 nm particles permeated tumor cells far from vessel centers [23]. In a recent report on 30 nm micellar nanomedicines, Cabral *et al*. expressed interest in the nature of particles less than 30 nm in diameter because they had found that nanoparticles of 30 nm were better able to penetrate poorly permeable hypovascular tumors than were larger nanoparticles [24]. A unique and exquisitely designed RNA nanotechnology, using phi29 DNA-packaging RNA, has attracted more and more attention due to its feasibility in cancer therapy [29, 30]. Various RNA moieties including siRNAs, ribozymes, antisense RNAs, aptamers and riboswitches can be easily conjugated into one single RNA nanoparticle [31–33]. Its size ranging from 10∼30 nm is optimal for systemic delivery, facilitating deep penetration through solid tumor [29, 30, 34–36]. Moreover, the nanoparticles exhibit minimum accumulation in liver, lungs, or any other normal organs due to its small size, consequently, having less immunogenicity [37–39]. Our system produces sCA in a similar range to packaging RNA nanoparticles. The diameter of over 5 nm was also convenient because it is known that particles < 5.5 nm are subject to capture in renal glomeruli [40].

We found that sCA nanoparticles had long-term stability in *ex vivo* mouse serum. As for *in vivo* half-life, Christie *et al*. determined blood circulation behavior using intravital real-time confocal laser scanning microscopy imaging of micelles prepared with fluorescently labeled siRNA, reporting that 2IT-95 micelles showed an over 2-fold improvement of circulation half-life and 1.7-fold increase in total fluorescence compared to naked-siRNA [41]. When we examined fluorescence intensity of sCA-siRNA by multiphoton live imaging at the mice ear microvasculature, we found that sCA showed a 2.9-fold improvement of circulation half-life of siRNA and 2.3-fold increase in AUC over 20 min compared to naked-siRNA. Therefore, *in vivo* circulation properties of sCA-siRNA were similar to those of micelles.

The zeta potential of sCA and sCA+0.5% albumin were found to be approximately-10 mV and-16 mV. This negative charge is helpful to avoid accumulation in the glomerular basement membrane due to charge repulsion [8]. Our finding that no sCA-siRNA accumulated in kidney is encouraging because this was a major problem with the PEGylated cyclodextrin-based polymer CALLAA-001 particles, the first siRNA nanoparticle-delivery system tested in clinical trial [42].

Owing to its small size, sCA should easily locate to tumor cells *in vivo* and enter the cells via endocytosis due to high transfection efficiency (Fig. 2). Our recent report has further revealed that carbonate apatite nanoparticles can be internalized by the cells in clathrin-dependent endocytosis [43]. IVIS imaging system and light sheet fluorescence microscopy clearly showed that sCA system could transfer siRNA to the tumor cells at 90 min ∼ 4 h. The great quantity of fluorescent siRNA in the cytoplasm of tumor cells at early time (Fig. 3C, S3C Fig.) indicates that sCA system is highly innovative. This early accumulation in tumor cells and quick release of siRNA by a pH-sensitive mechanism are considered vital features of sCA because intact siRNAs are expected to exert their roles soon after delivery and before they are damaged. For example, when intact siRNA remains in a target cell for an extended period it will be acidified and inactivated by lysosomes [25, 26]. Repeat treatment with sCA-survivin-siRNA indeed knocked down the survivin protein expression and induced apoptosis *in vivo*, indicating that delivered siRNA was functional in tumor cells. Consequently, the sCA system surpassed commercially available *in vivo* siRNA delivery systems in terms of their accumulation within tumors and their anti-tumor effect. In the latter experiments with survivin siRNA, we avoided to use the 5’-UGUGU-3’ sequence, because such sequence tends to stimulate innate cytokine responses (TNF-α, IL-6, INF-α, and INF-γ) in mammals via toll-like receptor (TLR) 7 and TLR8 [44]. We confirmed that innate cytokine responses (TNF-α, IL-6, INF-α, and INF-γ) were not observed in mice serum (S4 Fig.).

We administered a lower dose of survivin siRNA for anti-tumor proliferation experiments *in vivo* (a 15 μg of siRNA/injection, 7 times; total of 135 μg) relative to other systemic drug delivery systems for solid tumors [45–47], including cationic Lipofection complex (11.1-fold; 150 μg of siRNA/injection, 10 times), atelocollagen/siRNA (8.9-fold; 100 μg of siRNA/injection, 12 times), and clinically tested cyclodextrin-based polymer CALAA-01 (3.0–5.9-fold; 100–200 μg of siRNA/injection, 4 times). The requirement for a lower amount of siRNA is advantageous for avoiding hepatotoxicity. For example, Grimm *et al*. demonstrated shRNA-mediated liver toxicity due to saturation of the RNAi pathway [48]. Using sCA systems, we have thus far observed similar tumor inhibitory effects *in vivo* using 4 microRNAs and another siRNA (data not shown). For each of these, *in vitro* and even *in vivo* growth was reproducibly inhibited, and the observed tumor inhibitory effects were dose dependent.

Because of the very high accumulation of sCA-siRNA in tumor cells, we were concerned about toxicity if sCA accumulated much in normal organs. However, in contrast to tumor tissues, sCA was even less accumulated in lung, liver, spleen, and kidney as compared to IF and Atelo. We confirmed that repeat administrations of sCA alone to mice did not cause any hepatic damage or other problems related to change in body weight, blood chemistry, or histological findings in liver, spleen and kidney. Additional experiments administering 2.5- to 5-fold higher doses of sCA to mice proved to be safe as well. Kidney stones from patients with musculoskeletal anomalies or metabolic syndrome have been traditionally composed primarily of carbonate apatite [49, 50]. Because we conducted repeated systemic administration of sCA nanoparticles, the risk of kidney stone remained to be clarified. However, long term administration did not produce calculi in the urinary organs treated with sCA compared to the control ones (S5 Fig.), suggesting that injection of sCA nanoparticles does not cause calculi in urinary organs. Although the AST and ALT increased in one monkey (No. 1), isozyme analysis suggested that they were unlikely to be leaked from heart or liver, because cardiac muscle derived CPK-MB or liver associated LDH5 did not increase in the No. 1 monkey. Instead, skeletal muscle related CPK-MM and LDH1–2 elevated. These findings suggest that AST was derived from the skeletal muscles probably due to high stress of body restriction for 30 min during i.v. infusion. Hydroxyapatite [Ca_10_(PO_4_)_6_(OH)], a compound similar to sCA [Ca_10_(PO_4_)_6-X_(CO_3_)_X_(OH)_2_], has been used as a common material for dental use and orthopedic surgery in bone prosthesis applied to bone defects [51, 52] and our preliminary administration of sCA to *Cynomolgus* monkeys was executed in safety (Fig. 5D). These findings suggest safety of *in vivo* administration of sCA. However, we noticed nodular fluorescence of sCA-siRNA by 3D-IVIS (Fig. 3D) or minute green fluorescence spots of sCA-siRNA by fluorescence microscopy (S3D Fig.) in liver. We assume that these signals could be delivered from the larger nanoparticles around 653 nm, a minority constituent of sCA. Therefore, it would be important to purify the sCA nanoparticles uniformly before first administration in human.

## Conclusion

We have demonstrated an innovative *in vivo* siRNA delivery system targeting solid tumor models, which is characterized as 15 nm particle size in majority and considerable accumulation in the cytoplasm of tumor cells at early time after i.v. injection. Furthermore, this system is non-toxic in mice or monkeys. Our data suggest that sCA nanoparticle is a promising device for gene therapy of solid tumors.

## Materials and Methods

### Cell lines and reagents

Human colon cancer cell lines HCT116, HT29, the FaDu human head and neck cancer cell line and the 22Rv1 human prostate cancer cell line were purchased from the American Type Culture Collection. KM12SM was a kind gift from Prof. T. Minamoto [53] (Cancer Research Institute, Kanazawa University, Japan). HCT116, KM12SM, and FaDu cells were grown in DMEM, and HT29 and 22Rv1 cells were grown in RPMI supplemented with 10% fetal bovine serum (FBS). All cells were grown in 5% CO_2_ at 37°C. The isotope ^44^Ca was purchased from ISOFLEX.

### Animals

Seven-week-old BALB/cAJcl-nu/nu nude mice were purchased from CLEA Japan. All experiments using mice in this study were specifically approved by the Institutional Animal Care and Use Committee of Osaka University Graduate School of Medicine, and the Committee for the Ethics of Animal Experiments of Osaka University (Permit Number: 24–122–001, 23–023–001). All surgery was performed under sodium pentobarbital anesthesia, and all efforts were made to minimize suffering.

Osaka University Graduate School of Medicine does not have the enough supporting systems for *Cynomolgus* Monkeys experiments including house condition, feeding systems and specialized technicians. This is why we performed the *Cynomolgus* Monkeys experiments at BoZo Research Center where has expertise and the know-how in a wide range of studies including general toxicity, carcinogenicity, reproduction, development, irritation, sensitization etc. In this case, Osaka University dose not require us to apply the experiment protocols. All the approval of *Cynomolgus* Monkeys experiments were approved by the Institutional Animal Care and Use Committee (IACUC) of BoZo Research Center Inc. Three- to 5-year-old females (2.51 to 2.60 kg, n = 3) of *Cynomolgus* Monkeys (from Vietnam, producer: NAFOVANNY) were used at the animal testing facility of the Kannami Laboratory, Bozo Research Center Inc. This study was conducted in compliance with the following Regulations and Guidelines [54–56] for animal welfare. The IACUC of BoZo Research Center Inc. reviewed and approved the study. The permit number of the study is K130289. The animals were housed in an animal room (Room No. 401) which was set to maintain the temperature at 23 ± 5°C, relative humidity at 55 ± 25%, air ventilation at 9 to 15 times per hour and artificial lighting for 12 hours a day (07:00 to 19:00). They were housed individually in stainless-steel cages (W 750 × D 750 × H 700 mm). Animals were supplied with 150 g of pelleted diet PS for monkeys (Oriental Yeast Co., Ltd., Japan) once every day at a set time between 12:00 pm and 17:00 pm, and remaining diet was removed between 08:00 am and 10:00 am on the next day. They were allowed ad libitum tap water (Fujimi Water Union, Japan) via an automatic water supply system. A metal mirror was provided for each cage as an environmental enrichment. In the present study, physical restrain duration was approximately 30 min. Close observation was performed during the restraint period, and behavior, unusual responses such as aggression and timidity were checked. After completion of study, the animals were excluded from the study and kept continuously at the facility.

### siRNA

6-FAM labeled control siRNA was purchased from KOKEN and was used for *in vitro* and *in vivo* fluorescence microscopy surveys and flow cytometric analysis; control siRNA: 5’-AUCCGCGCGAUAGUACGUAUUdTdT-3’.

Survivin siRNA and control siRNA for *in vitro* use were purchased from QIAGEN. Survivin siRNA for *in vivo* use, with or without 6-FAM conjugation, was purchased from KOKEN. The sequence of the survivin siRNA used was 5’-GCAUUCGUCCGGUUGCGCUdTdT-3’.

For IVIS spectrum CT analysis, Alexa Flour 750 labeled control siRNA was purchased from GeneDesign; control siRNA: 5’-UACGUACUAUCGCGCGGAU-3’.

### Production of sCA

For *in vitro* use, 4 μL of 1 M CaCl_2_ was mixed with 2 μg of siRNA in 1 mL of serum-free bicarbonate (44 mM)-buffered DMEM medium (pH 7.5) followed by incubation at 37°C for 30 min. For *in vivo* experiments, an inorganic solution (NaHCO_3_; 44 mM, NaH_2_PO_4_; 0.9 mM, CaCl_2_; 1.8 mM, pH 7.5) was used instead of the DMEM medium solution. The solution was centrifuged at 12,000 rpm for 3 min, and the pellet was dissolved with saline containing 0.5% albumin. The products in the solution were sonicated (38 kHz, 80 W) in a water bath for 10 min to generate sCA+0.5% albumin, which were intravenously injected within 10 min.

### Assays for nanoparticle features

The distribution of particle size was measured using Fiber-Optical Dynamic Light-Scattering Spectrophotometer FDLS-3000 (Otsuka Electronics, Osaka, Japan) equipped with a 532 nm diode laser. The measurement was carried out by a scattering angle of 90° at 25°C. Size distributions were determined by cumulant and histogram analysis of DLS data using the software provided by the manufacturer. The morphology and size distribution of the sCA nanoparticles was further analyzed by laser microscope (OLS-4100, Shimadzu) and atomic force microscopy using a scanning probe microscope (SPM-9500, Shimadzu) in dynamic mode and equipped with a microcantilever (OMCL-AC240TS-R3, Olympus). Optical density (OD) of the samples at 655 nm was measured spectrophotometrically at different pH using a microplate reader (680 XR, Bio-Rad). The zeta potential of the particles was measured by Laser Doppler Micro-electrophoresis (Zetasizer Nano Z, Malvern) at 25°C for 30 sec.

### *Ex vivo* half-life of naked-siRNA or sCA-siRNA

Forty μg of naked-siRNA or sCA-siRNA was incubated in 2 mL mouse serum at 37°C for various time periods. The serum was centrifuged to gather sCA-siRNA nanoparticles, and the pellet was dissolved in 2 mL 0.02 M EDTA. Collected naked-siRNA or sCA-siRNA was mixed with loading dye (Thermo Scientific), and loaded in a 4.5% NuSieve GTG agarose gel.

### Inductively coupled plasma mass spectrometry analysis

Blood sample (0.1 g) was digested with 1 mL H_2_O_2_ and 2 mL HNO_3_ using microwave digestion equipment (MILESTONE ETHOS1). The test solution was prepared by adding 20 mL DW to the digested blood sample. The concentration of ^44^Ca in the test solution (10 mL) with 0.1 mL ^71^Ga (5 μg/mL) as internal standard was analyzed using inductively coupled plasma mass spectrometry (Agilent 7500ce).

### Flow cytometric analysis

Cells were harvested with a solution of 2.5 g/L Trypsin and 1 mmol/L EDTA (Nacalai Tesque) and were subjected to flow cytometric analysis using a BD FACS Aria II instrument (BD Biosciences). A total of 10,000 events were recorded for each analysis, and the data were analyzed using BD FACS Diva software (BD Biosciences).

### Confocal laser microscopy

Cells were fixed in 4% paraformaldehyde and were mounted with ProLong Gold antifade reagent with DAPI (Invitrogen). The samples were analyzed by confocal laser scanning microscope (FV1000-D, Olympus).

### Cell proliferation assay

Cells were uniformly seeded into 96-well plates (1 × 10^4^ cells/well). Cell viability was examined at 48 and 72 h by WST-8 assay (Dojindo). Absorbance was assessed spectrophotometrically by microplate reader (680 XR, Bio-Rad) with wavelength of 450 nm.

### Western blot analysis

Tumor tissues were homogenized on ice with a homogenizer (Tissue Lyser, QIAGEN), or harvested cells were lysed with RIPA buffer (Thermo Scientific). Protein samples (30 μg) were separated by SDS-PAGE followed by electroblotting onto a polyvinylidene fluoride membrane. After blocking with Blocking One (Nacalai Tesque), the membrane was incubated overnight with primary antibodies against survivin (Novus Biologicals) and actin (Sigma), both at 1:1000 dilutions. The protein bands were detected using the Amersham ECL Detection System (Amersham Biosciences).

### Imaging systems

Mice were imaged under anesthesia using the IVIS Spectrum CT instrument (PerkinElmer) in fluorescence mode for 3D imaging analysis or IVIS lumina for *ex vivo* imaging. All data were analyzed using Living Image 4.3. Frozen sections 20-μm thick were evaluated with a fluorescence microscope (BZ-9000, KEYENCE). The fluorescence intensity/area of each sample was measured by the application software (BZ-H1C). Frozen sections 1-mm thick were embedded in 1% low melting point agarose gel and were evaluated with a light sheet fluorescence microscope (Lightsheet Z.1, Carl Zeiss Microscopy). In order to measure the fluorescent siRNA for determining the *in vivo* half-lives of sCA-siRNA and naked-siRNA following tail-vein intravenous injection (80 μg siRNA for naked-siRNA, 40 μg siRNA for sCA-siRNA), and to visualize the extravasation of the nanoparticles from the tumor vessels, the multiphoton live imaging was performed on animals under anesthesia. The imaging system was composed of a multiphoton microscope (SP5; Leica) driven by a laser (Mai-Tai HP Ti: Sapphire; Spectraphysics) tuned to 900 nm, and an upright microscope (DM6000B; Leica) equipped with a 20x water immersion objective (HCX APO, N.A. 1.0; Leica).

### Fluorescence immunostaining

The frozen sections were incubated overnight with the anti-survivin polyclonal antibody (Novus Biologicals) at the concentration of 1:1000. After incubating with the secondary antibody (goat anti-rabbit IgG Alexa Fluor 633, Invitrogen), the samples were mounted with ProLong Gold antifade reagent with DAPI (Invitrogen).

### TUNEL assay

The detection of apoptotic cells was performed using the DeadEnd Fluorometric TUNEL System (Promega) as described in the manufacturer’s protocol. The fragmented DNA of apoptotic cells is detected by catalytically incorporating fluorescein-12-dUTP at 3’-OH DNA ends.

### Tumor models

Human colon cancer HCT116, HT29 cells, or human head and neck cancer FaDu (5.0 × 10^6^ cells/tumor) were inoculated subcutaneously in both the left and right flanks of mice to establish solid tumors.

### Anti-tumor activity assay

Systemic injection started when the HCT116 tumor volume reached approximately 80 mm^3^, with the administration of 15 μg of control-siRNA or survivin-siRNA delivered by IF, Atelo, or sCA. Mice were subsequently treated on days 0, 2, 4, 7, 9, 11, 14, 16, and 18. Anti-tumor activity was evaluated in terms of tumor size (n = 10 for each group), which was estimated using the following equation: V = a × b^2^ /2, where a and b represent the major and minor axes of the tumor, respectively. Invivofectamine 2.0 reagent (Invitrogen) and AteloGene (KOKEN) were used as systemic *in vivo* siRNA delivery systems for comparison with sCA.

### Cytokine ELISA

Survivin siRNA (40 μg) encapsulated in IF, Atelo, or sCA was administered intravenously. At 6 and 12 h, blood samples were collected and processed as plasma for cytokine analysis. The levels of mouse serum cytokines were quantified using sandwich ELISA kits to detect mouse INF-α (PBL Biomedical), IL-6, TNF-α, and INF-γ (R&D Systems).

### Blood chemistry tests and calculi examination

Blood sample was (100 μL) was applied to the multirotor 2 (VetScan) and then analyzed by VetScan VS2 for chemistry tests. As for calculi examination, the harvested kidney, urinary duct and bladder were investigated using X-ray photography (70kV, 7mA, 0.08sec, DentNavi) and stereomicroscope (Leica).

### Statistical analysis

Statistical analysis was carried out using the JMP10 program (SAS Institute). Data are expressed as the mean ± SD and analyzed with the Wilcoxon rank test. The results were considered statistically significant if two-tailed *P*-values were less than 0.05.

## Supporting Information

S1 ARRIVE Checklist (PDF)

S1 Fig (A) AFM image of sCA nanoparticles with cross-section profile is shown. The nanoparticle size ranged from 5 to 30 nm (mean ± SD: 10.50 ± 5.01 nm). (B) After sonication, the sCA nanoparticles precipitated within 1 min (black arrow). Addition of 0.5% albumin during sonication prevented precipitation at 10 min after sonication (sCA+0.5% alb). This effect was not achieved by addition of 0.5% transferrin (sCA+0.5%TF).
(TIF)

S2 Fig Cellular uptake of fluorescently labeled siRNA by Lp or sCA in cancer cells (FaDu, HT29, KM12SM and 22Rv1) by flow cytometry and fluorescence microscopy analyses. (TIF)

S3 Fig (A) Early uptake of sCA-siRNA at 3, 9, and 16 h. (B) Early uptake of sCA-siRNA in FaDu model at 1.5, 3, and 9 h. *P = 0.0022; **P = 0.0034; ***P = 0.0024 (n = 6∼8 tumors, Wilcoxon rank test). (C) Light sheet fluorescence microscopy images from continuous sections of the sCA-siRNA treated tumors. Scale bar, 10 μm. Fluorescent siRNA signals accumulated throughout the cytoplasm of tumor cells. (D)(E)(F) Detection of 6-FAM labeled siRNA distribution by fluorescence microscopy in normal tissues. (D) View of liver 4 and 12 h after intravenous injection, treated with IF-siRNA, Atelo-siRNA or sCA-siRNA. Scale bar, 50 μm. (E) Spleen at 4 and 12 h. Scale bar, 50 μm. (F) Kidney at 4 and 12 h. Scale bar, 50 μm.
(TIF)

S4 Fig Immune response by measuring cytokine levels including TNF-α, IL-6, INF-α, and INF-γ. Survivin siRNA (40 μg) encapsulated in IF, Atelo, or sCA was administered intravenously. At 6 and 12 h, blood samples were collected and processed as plasma for cytokine analysis. The levels of mouse serum cytokines were quantified using sandwich ELISA kits to detect mouse INF-α, IL-6, TNF-α, and INF-γ. No significant induction of the cytokines was noted.
(TIF)

S5 Fig Investigation of the existence of calculi in kidney, urinary duct and bladder using X-ray photography and stereomicroscope. The X-ray photography analysis was performed with a 15 cm metal rod as radiopaque standard.
(TIF)

S6 Fig Aggregation of the original carbonate apatite. (A) Aggregated carbonate apatite nanoparticles on an HCT116 cell observed by scanning electron microscopy, Scale bar: 4 μm. (B) Atomic force microscopy shows that individual nanoparticles actually form into large aggregations, the size of which can reach >1000 nm. Scale bar: 1 μm.
(TIF)

S7 Fig siRNA nanoparticle delivery systems classified by size. The nanoparticles currently available to carry siRNA are liposomes, micelles, polymers, and atelocollagen, with sizes in the range of 45–300 nm. sCA and RNA packaging nanoparticles are the smallest classes of nanoparticles.
(TIF)

S1 Table Test items and schedule of non-human primate toxicity study. (TIF)

S2 Table Items and methods of examination on cumulative urine samples. (TIF)

S3 Table Items and methods of examination on blood samples. (TIF)
