# Supplementary figures and images for: Innovative Delivery of siRNA to Solid Tumors by Super Carbonate Apatite
Source: PLoS One. 2015 Mar 4;10(3):e0116022. doi: 10.1371/journal.pone.0116022 (PMC4349808; doi:10.1371/journal.pone.0116022)

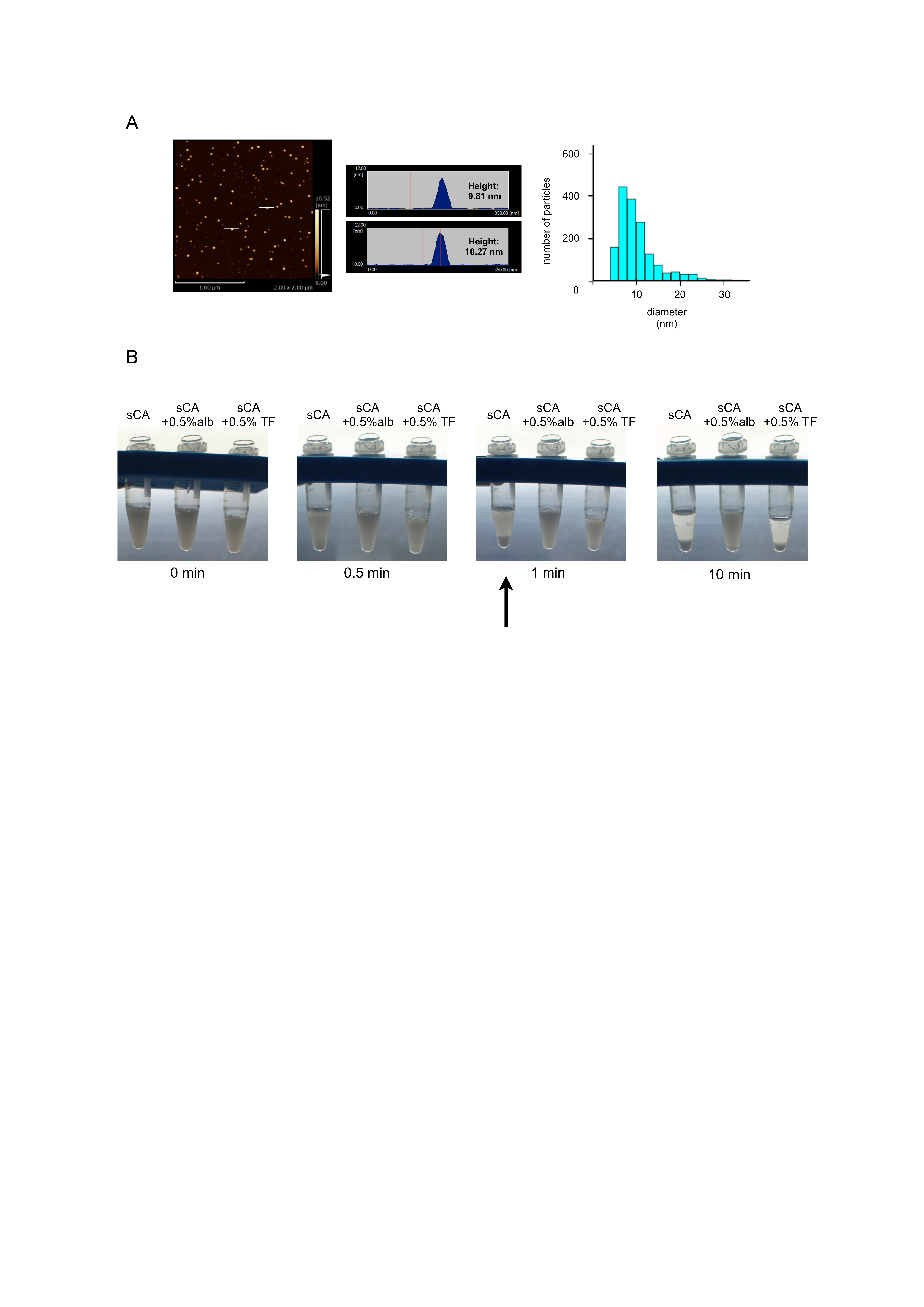

Supplement: S1 Fig — The nanoparticle size ranged from 5 to 30 nm (mean ± SD: 10.50 ± 5.01 nm). (B) After sonication, the sCA nanoparticles precipitated within 1 min (black arrow). Addition of 0.5% albumin during sonication prevented precipitation at 10 min after sonication (sCA+0.5% alb). This effect was not achieved by addition of 0.5% transferrin (sCA+0.5%TF). (TIF) [file pone.0116022.s002.tif]

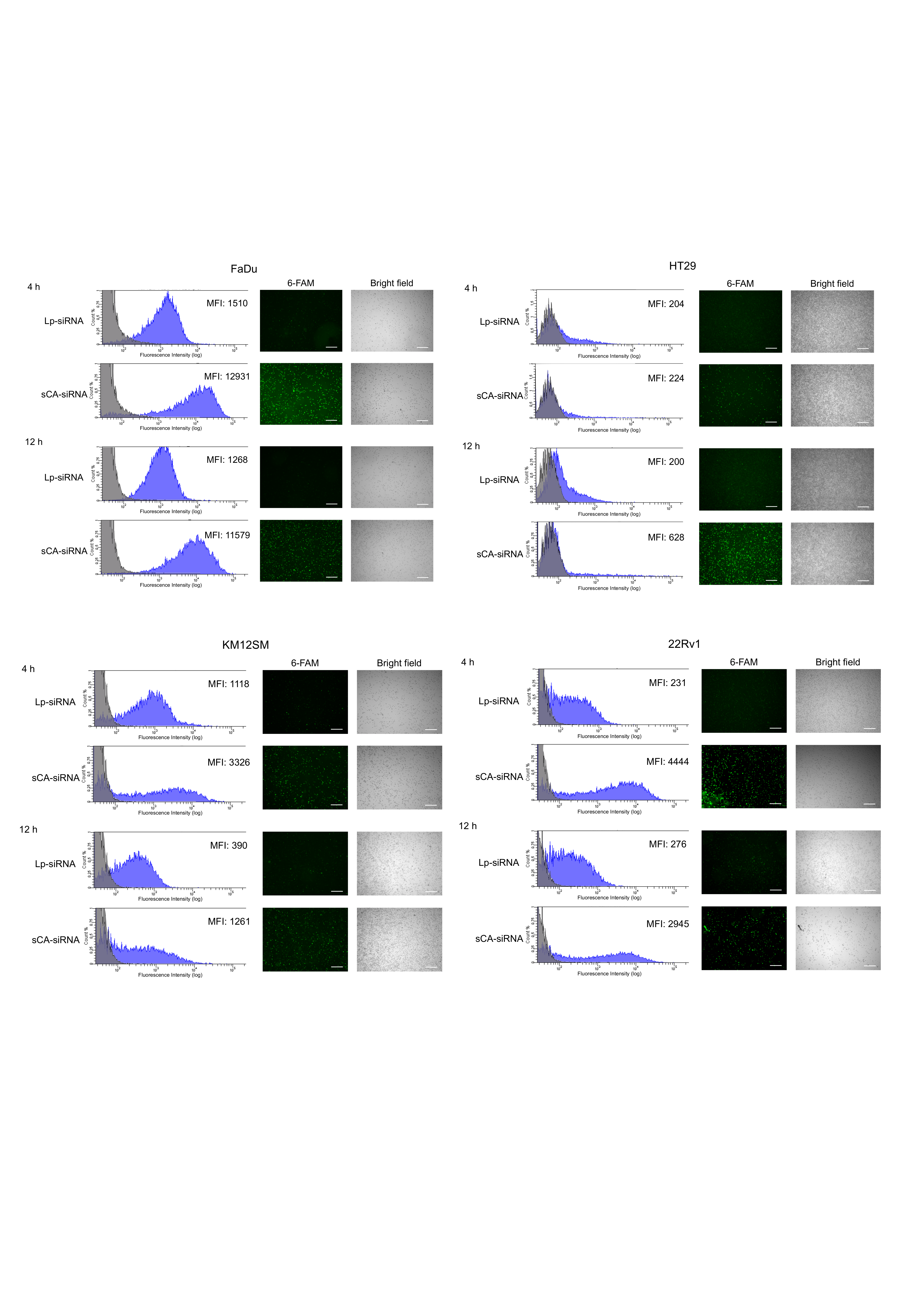

Supplement: S2 Fig — (TIF) [file pone.0116022.s003.tif]

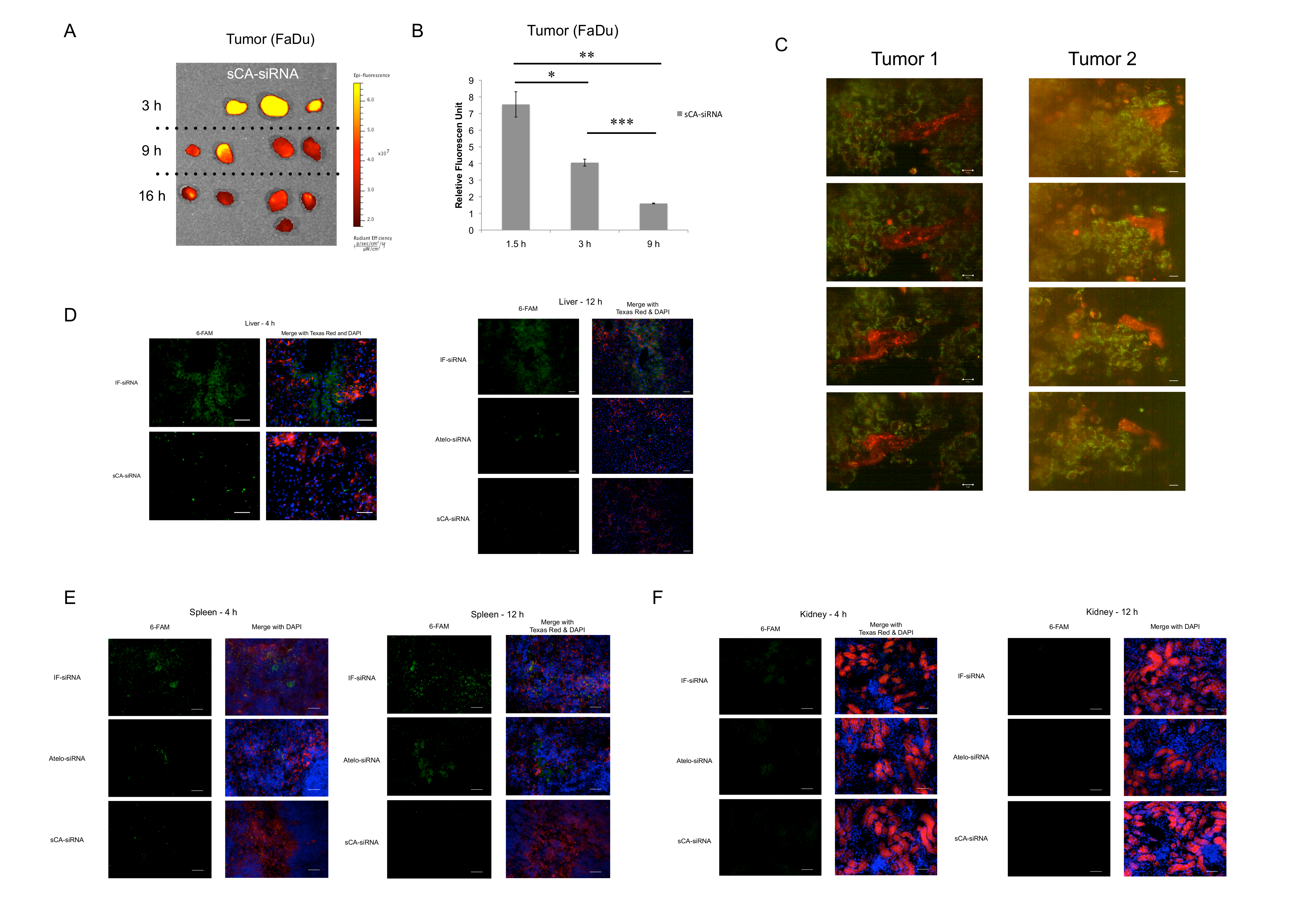

Supplement: S3 Fig — (B) Early uptake of sCA-siRNA in FaDu model at 1.5, 3, and 9 h. *P = 0.0022; **P = 0.0034; ***P = 0.0024 (n = 6∼8 tumors, Wilcoxon rank test). (C) Light sheet fluorescence microscopy images from continuous sections of the sCA-siRNA treated tumors. Scale bar, 10 μm. Fluorescent siRNA signals accumulated throughout the cytoplasm of tumor cells. (D)(E)(F) Detection of 6-FAM labeled siRNA distribution by fluorescence microscopy in normal tissues. (D) View of liver 4 and 12 h after intravenous injection, treated with IF-siRNA, Atelo-siRNA or sCA-siRNA. Scale bar, 50 μm. (E) Spleen at 4 and 12 h. Scale bar, 50 μm. (F) Kidney at 4 and 12 h. Scale bar, 50 μm. (TIF) [file pone.0116022.s004.tif]

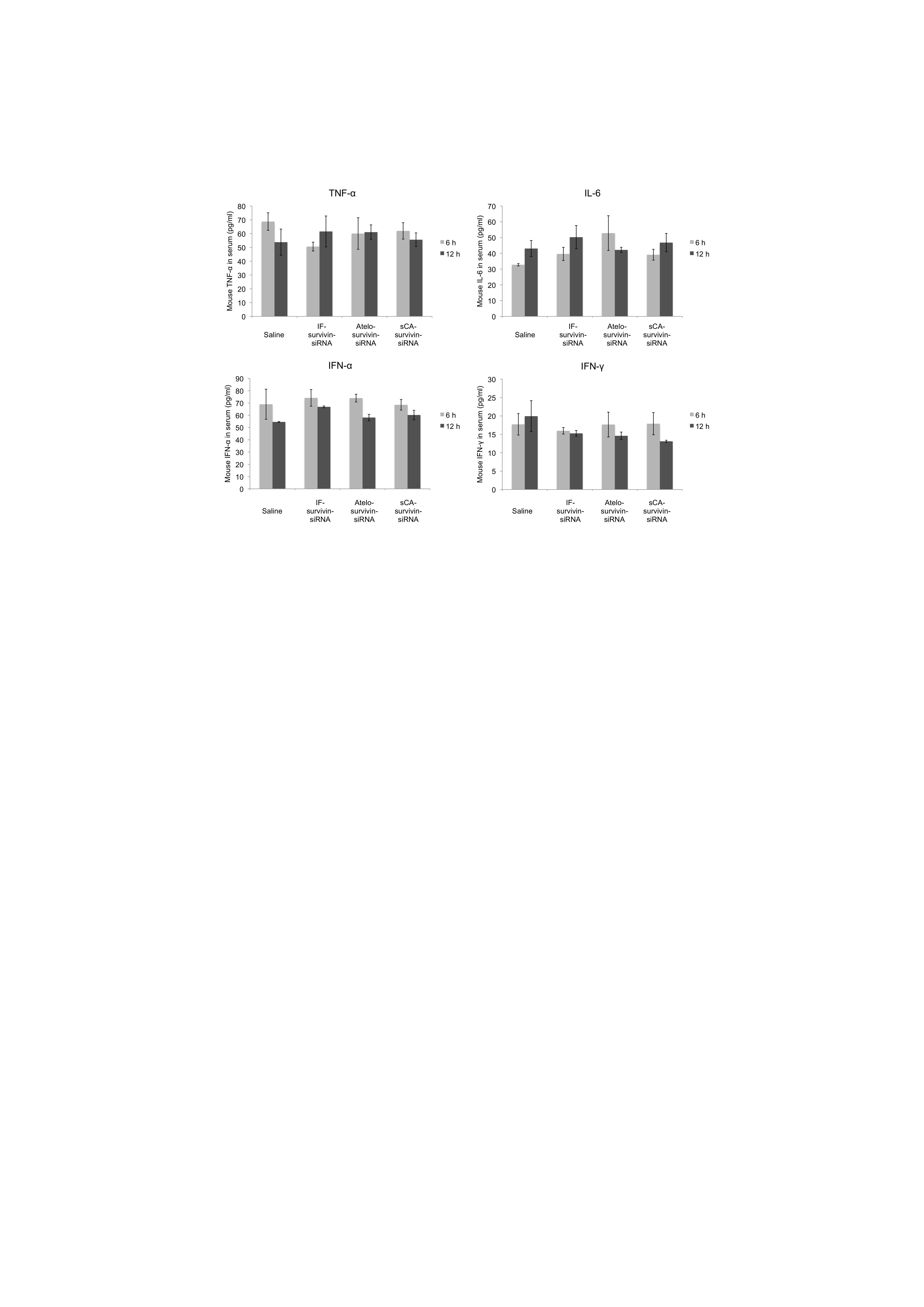

Supplement: S4 Fig — At 6 and 12 h, blood samples were collected and processed as plasma for cytokine analysis. The levels of mouse serum cytokines were quantified using sandwich ELISA kits to detect mouse INF-α, IL-6, TNF-α, and INF-γ. No significant induction of the cytokines was noted. (TIF) [file pone.0116022.s005.tif]

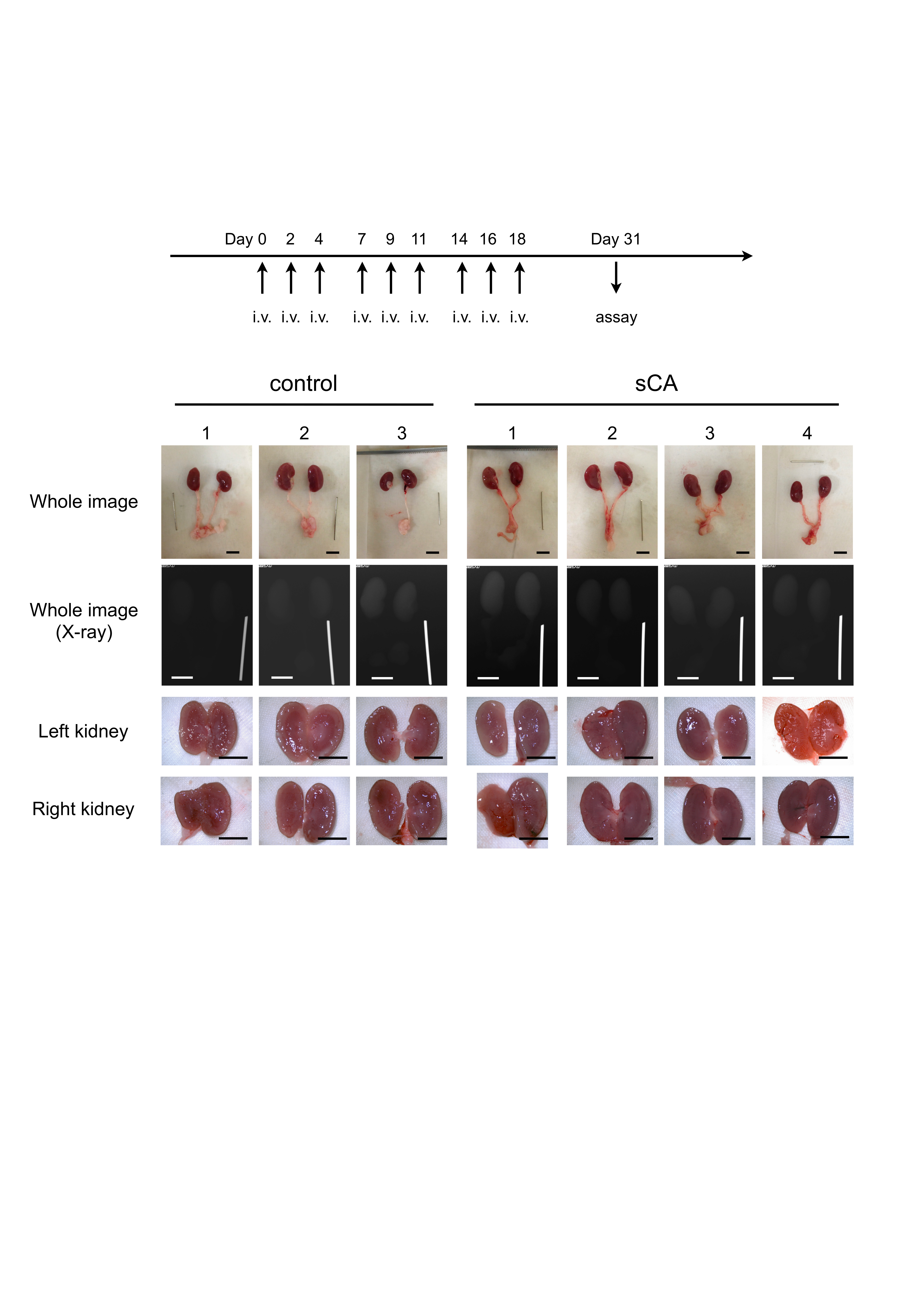

Supplement: S5 Fig — The X-ray photography analysis was performed with a 15 cm metal rod as radiopaque standard. (TIF) [file pone.0116022.s006.tif]

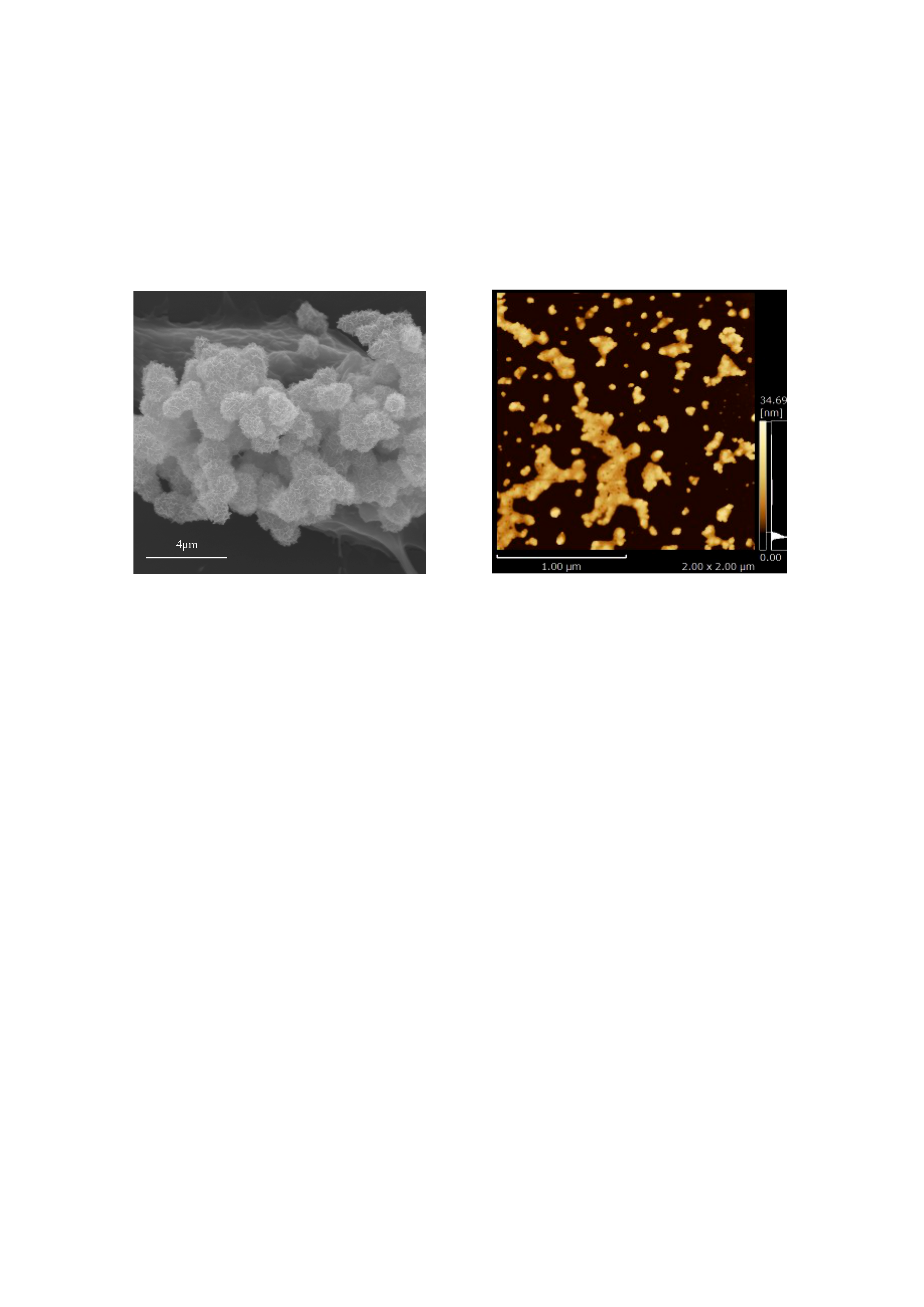

Supplement: S6 Fig — (A) Aggregated carbonate apatite nanoparticles on an HCT116 cell observed by scanning electron microscopy, Scale bar: 4 μm. (B) Atomic force microscopy shows that individual nanoparticles actually form into large aggregations, the size of which can reach >1000 nm. Scale bar: 1 μm. (TIF) [file pone.0116022.s007.tif]

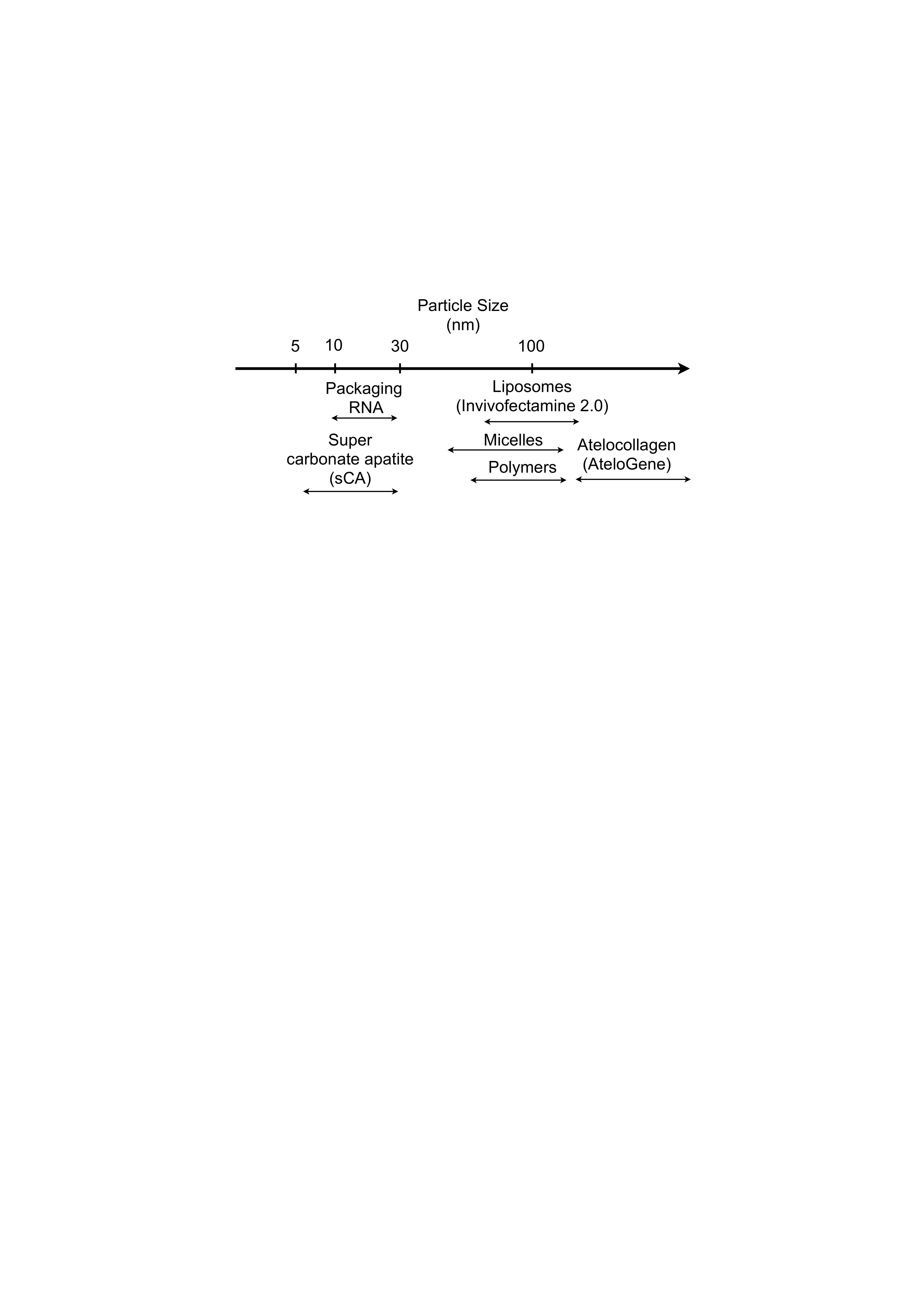

Supplement: S7 Fig — The nanoparticles currently available to carry siRNA are liposomes, micelles, polymers, and atelocollagen, with sizes in the range of 45–300 nm. sCA and RNA packaging nanoparticles are the smallest classes of nanoparticles. (TIF) [file pone.0116022.s008.tif]

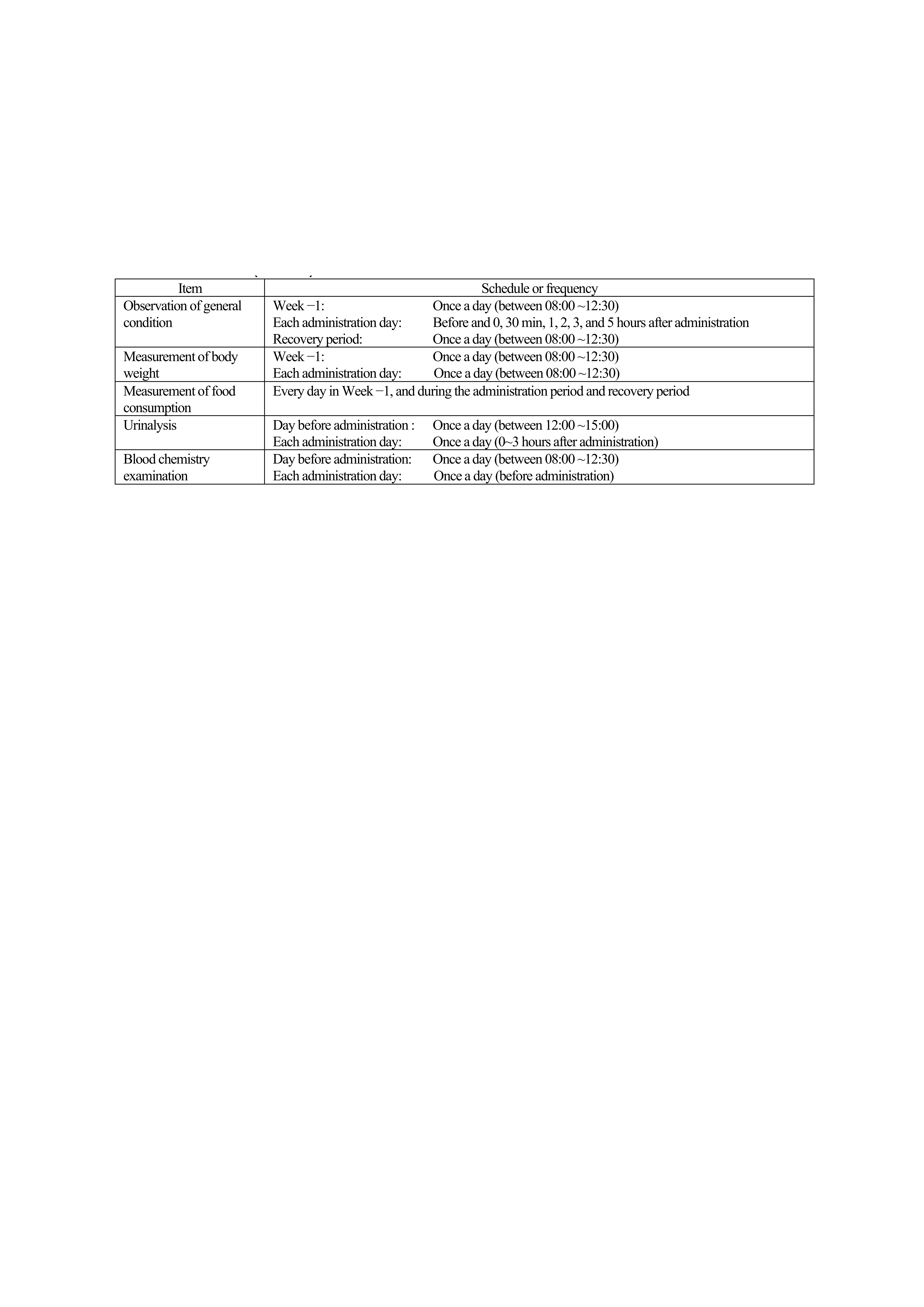

Supplement: S1 Table — (TIF) [file pone.0116022.s009.tif]

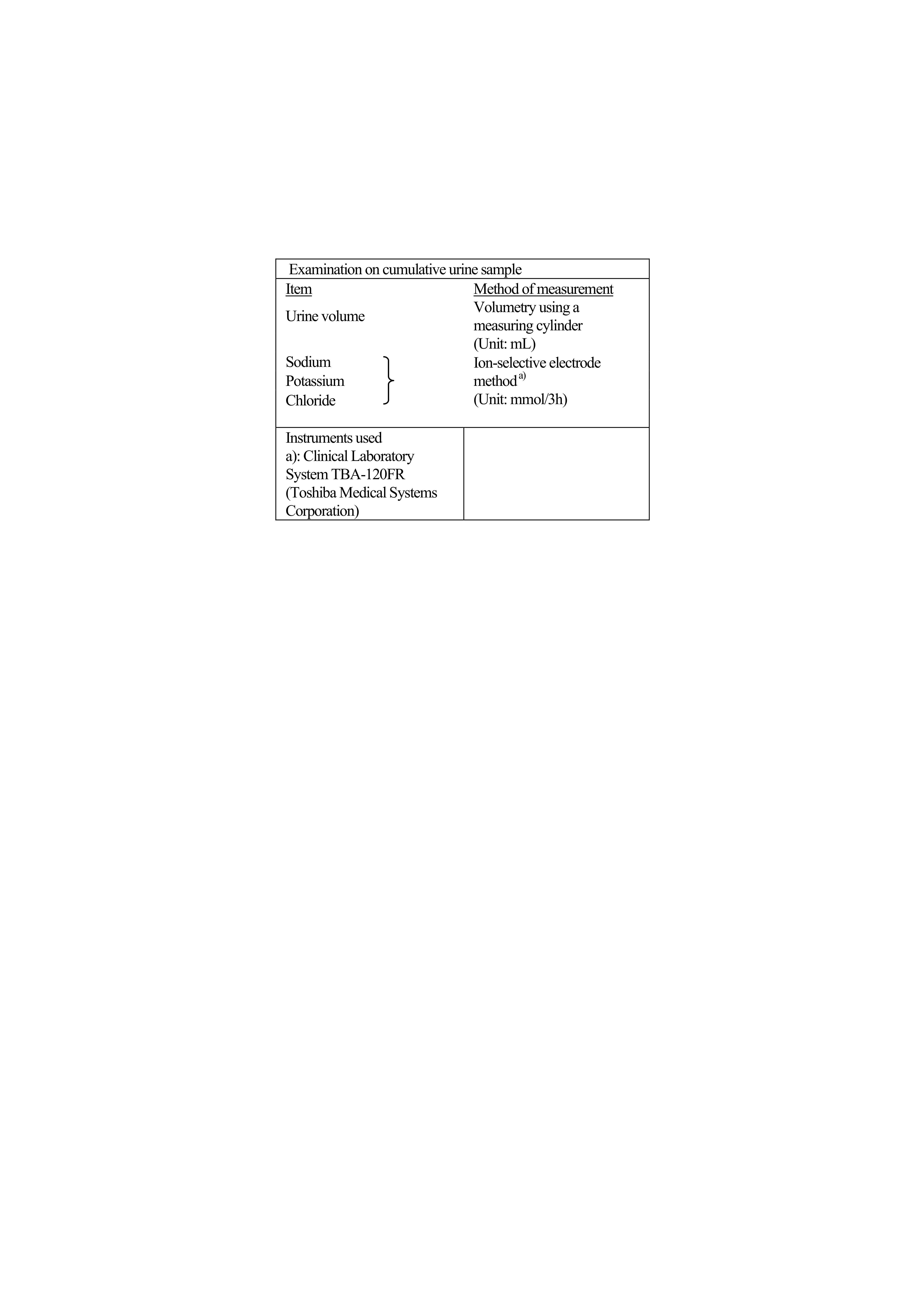

Supplement: S2 Table — (TIF) [file pone.0116022.s010.tif]

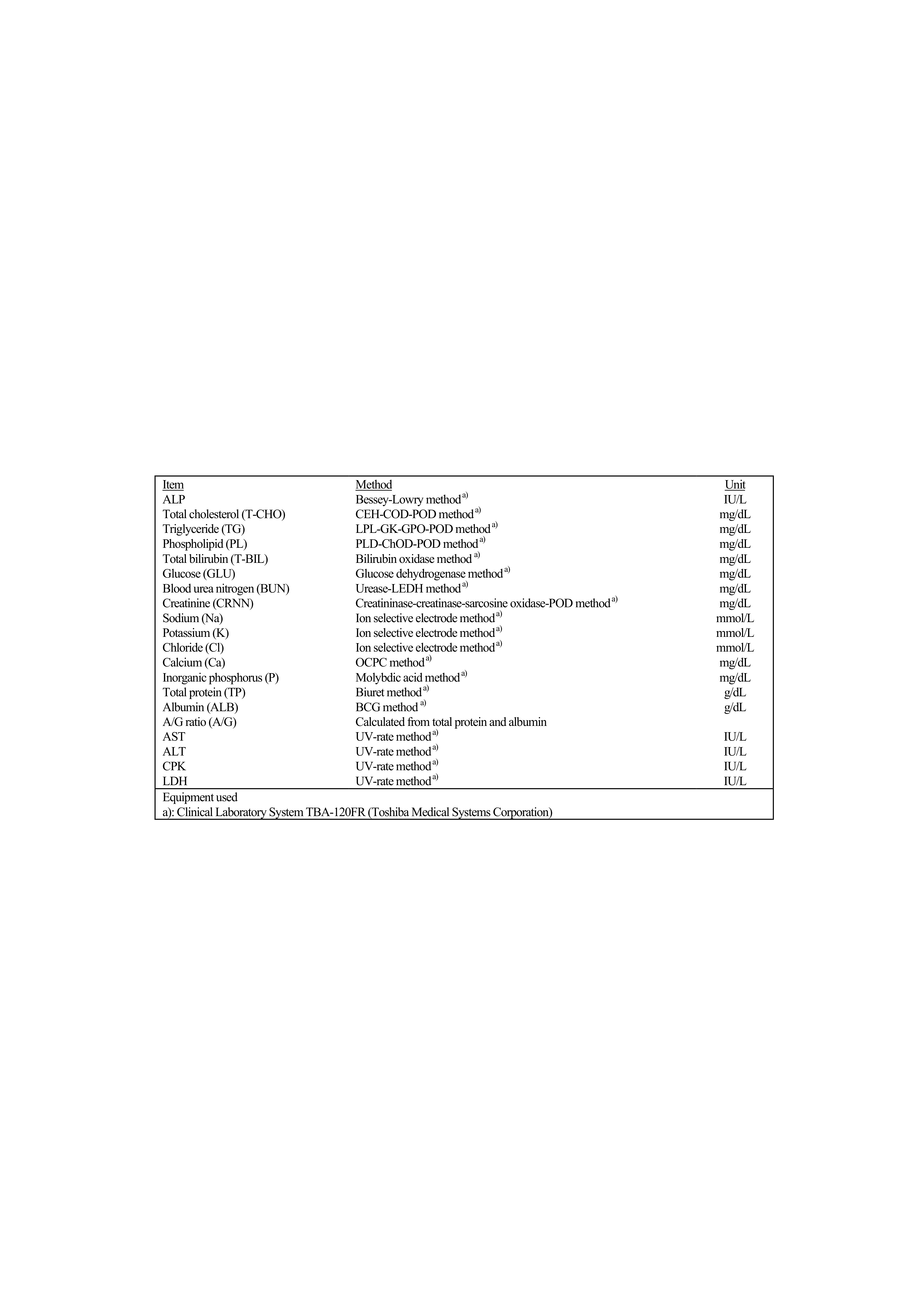

Supplement: S3 Table — (TIF) [file pone.0116022.s011.tif]
